# Coding and Noncoding Variation in *LRRK2* and Parkinson's Disease Risk

**DOI:** 10.1002/mds.28787

**Published:** 2021-09-20

**Authors:** Julie Lake, Xylena Reed, Rebekah G. Langston, Mike A. Nalls, Ziv Gan‐Or, Mark R. Cookson, Andrew B. Singleton, Cornelis Blauwendraat, Hampton L. Leonard, International Parkinson's Disease Genomics Consortium (IPDGC)

**Affiliations:** ^1^ Laboratory of Neurogenetics, National Institute on Aging National Institutes of Health Bethesda Maryland USA; ^2^ Center for Alzheimer's and Related Dementias National Institutes of Health Bethesda Maryland USA; ^3^ Data Tecnica International Glen Echo Maryland USA; ^4^ Montreal Neurological Institute McGill University Montréal Quebec Canada; ^5^ Department of Human Genetics McGill University Montréal Quebec Canada; ^6^ Department of Neurology and Neurosurgery McGill University Montréal Quebec Canada; ^7^ German Center for Neurodegenerative Diseases (DZNE) Tübingen Germany

**Keywords:** Parkinson's disease, genetics, association, LRRK2

## Abstract

**Background:**

The leucine‐rich repeat kinase 2 (*LRRK2*) gene harbors both rare highly damaging missense variants (eg, p.G2019S) and common noncoding variants (eg, rs76904798) with lower effect sizes that are associated with Parkinson's disease (PD) risk.

**Objectives:**

This study aimed to investigate in a large meta‐analysis whether the *LRRK2* Genome‐Wide Association Study (GWAS) signal represented by rs76904798 is independently associated with PD risk from *LRRK2* coding variation and whether complex linkage disequilibrium structures with p.G2019S and the 5′ noncoding haplotype account for the association of *LRRK2* coding variants.

**Methods:**

We performed a meta‐analysis using imputed genotypes from 17,838 patients, 13,404 proxy patients, and 173,639 healthy controls of European ancestry. We excluded carriers of p.G2019S and/or rs76904798 to clarify the role of *LRRK2* coding variation in mediating disease risk and excluded carriers of relatively rare *LRRK2* coding variants to assess the independence of rs76904798. We also investigated the co‐inheritance of *LRRK2* coding variants with p.G2019S, rs76904798, and p.N2081D.

**Results:**

*LRRK2* rs76904798 remained significantly associated with PD after excluding the carriers of relatively rare *LRRK2* coding variants. *LRRK2* p.R1514Q and p.N2081D were frequently co‐inherited with rs76904798, and the allele distribution of p.S1647T significantly changed among patients after removing rs76904798 carriers.

**Conclusions:**

These data suggest that the *LRRK2* coding variants previously related to PD (p.N551K, p.R1398H, p.M1646T, and p.N2081D) do not drive the 5′ noncoding GWAS signal. These data, however, do not preclude the independent association of the haplotype p.N551K‐p.R1398H and p.M1646T with altered disease risk. © 2021 The Authors. *Movement Disorders* published by Wiley Periodicals LLC on behalf of International Parkinson Movement Disorder Society. This article has been contributed to by US Government employees and their work is in the public domain in the USA.

The leucine‐rich repeat kinase 2 (*LRRK2*) gene has been a focus of Parkinson's disease (PD) research since the discovery that pathogenic mutations in the gene are related to autosomal‐dominant PD.^1^, ^2^ The most common pathogenic mutation, p.G2019S, has a relatively high frequency in Ashkenazi Jews and North African Arabs and is also found in about 1% of Europeans with PD.^3^ Although the underlying mechanism of toxicity is not fully elucidated, the p.G2019S mutation has been shown to increase LRRK2 kinase activity, which is considered to cause a toxic gain of function.^4^ Hyperactive mutations in *LRRK2* have been shown to impact lysosomal and endocytic regulation by increasing *LRRK2* recruitment to lysosomes.^5^ Some neuropathological changes and clinical features caused by LRRK2‐PD are similar to those of idiopathic PD, suggesting that there could be common mechanisms of pathogenesis and potentially common therapies.^6^

Several studies have nominated *LRRK2* missense variants as either causal for or associated with an increased risk of PD in individuals of Asian (eg, p.A419V, p.R1628P, and p.G2385R), Arab‐Berber (eg, p.Y2189C), and European ancestries (eg, p.M1646T, p.N2081D, and p.R1441C/G/H).^7^, ^8^, ^9^, ^10^, ^11^, ^12^, ^13^, ^14^ A common protective haplotype (p.N551K‐p.R1398H‐p.K1423K) has also been observed in individuals of both Asian and European ancestries.^7^ In addition, common noncoding variation (rs76904798, odds ratio [OR]: 1.15, 95% confidence interval [CI]: 1.13–1.18, *P* = 1.52e‐28) upstream of *LRRK2* is associated with increased PD risk, an effect that is independent of the p.G2019S mutation.^15^

Although the exact causal variant at the 5′ GWAS signal of *LRRK2* is unknown,^16^, ^17^ it has been shown in a relatively small cohort of 1381 PD patients and 1328 controls that the 5′ signal had a low degree of correlation with known coding susceptibility variants, including p.M1646T and protective haplotype p.N551K‐R1398H‐K1423K, indicating the independence of this GWAS signal.^18^ The independence of this signal from *LRRK2* coding variation suggests that changes in the expression or splicing of *LRRK2* could mediate PD risk. There is evidence that the GWAS‐nominated noncoding variation tagged by rs76904798 could affect *LRRK2* expression. The allele rs76904798‐T has been associated with increased *LRRK2* expression in monocytes,^19^ in monocyte‐derived microglia‐like cells,^20^ and in both human brain microglia and induced pluripotent stem cell (iPSC)‐derived models.^21^ The same allele has also been associated with faster development of motor symptoms.^22^ To further investigate the pattern of association at the *LRRK2* locus and determine in a significantly larger data set whether rs76904798 is independently associated with PD from *LRRK2* coding variation, we performed a conditional association analysis using individual‐level genotype data from 17,838 PD patients, 13,404 proxy patients (ie, individuals with a first‐degree relative who has PD but does not have PD themselves), and 173,639 healthy controls of European ancestry.

## Patients and Methods

### Genotyping Data

#### International Parkinson's Disease Genomics Consortium Genotyping Data

Genotype data were obtained as previously described.^15^ Only data sets with participants who had high‐quality imputation scores (R^2^ > 0.8) for p.G2019S and the 5′ noncoding variant rs76904798 were included. Quality control parameters can be found in the Supplementary Information. In total, 13 data sets were included from the International Parkinson's Disease Genomics Consortium (IPDGC), with individual‐level genotypes from 16,309 PD patients and 17,705 healthy controls of European ancestry (Table S1). Following quality filtering, data sets were imputed using the Haplotype Reference Consortium imputation panel r1.1 2016 through the Michigan imputation server with default settings with phasing using the EAGLE v2.3 option.^23^ Genotypes were filtered for imputation quality R^2^ > 0.8, with the exception of rs10847864 (*HIP1R* GWAS signal) that was included as an independent signal on chromosome 12 (located ~82 mb upstream of *LRRK2*) despite slightly lower imputation quality in the Myers‐Foroud (MF)^24^ and Spanish Parkinson's (IPDGC) cohorts (SPAIN3 and SPAIN4) (Table S2). Principal components (PCs) were calculated as described in the Supplementary Information.

#### UK Biobank Data

The UK Biobank is a large study of ~500,000 individuals from the United Kingdom with a variety of phenotypic information, including information on PD status such as ICD‐10 designation and self‐report.^25^ PD patients were identified using field code 42033, and proxy patients were included based on their paternal PD status (data field 20107) and maternal PD status (data field 20110). We have previously shown that these proxy patients share genetic risk with PD patients.^15^ Genotype data were obtained as previously described^15^ and split into case–control and proxy–control data sets for separate GWAS. Genotypes were filtered for imputation quality R^2^ > 0.8. Quality control parameters are available in the Supplementary Information. After quality control, the case–control data set consisted of individual‐level genotypes from 1529 patients and 15,279 healthy controls, with the controls selected randomly from the pool of nonaffected, nonproxy individuals in the biobank. The proxy–control data set consisted of 13,404 proxy patients and 140,655 healthy controls (Table S1). PCs were calculated as described in the Supplementary Information.

### Association Analyses

Summary statistics were generated for each study using a logistic regression model on imputed genotypes from chromosome 12, followed by Firth regression when logistic regression failed to converge. IPDGC data were adjusted for biological sex, age, and the first five PCs representing population substructure. Age at onset was used for PD cases and age at study for healthy controls, except in the Vance (dbGap phs000394) and MF^24^ cohorts due to missing data. The UK Biobank data were adjusted for biological sex, age at recruitment, Townsend, and the first five PCs. Genome‐wide association study by proxy was carried out as previously described on UK Biobank proxy‐case data^15^ because these individuals are at‐risk but not true cases. A fixed‐effects model was fitted using METAL v2018‐08‐28 under default settings^26^ to combine summary statistics across the 13 cohorts from IPDGC and the UK Biobank case–control and proxy‐control data sets. After GWAS filtering of multi‐allelic variants, regional plots of the meta‐analysis results were made around the *LRRK2* gene using LocusZoom v1.3.^27^ Forest plots were generated using the metafor package in R. A summary of the association results for the *LRRK2* variants examined in this study is presented in Table 2 and Table S3. We included an independent association signal on chromosome 12 at the *HIP1R* locus (rs10847864), identified in a previous PD meta‐analysis,^15^ in all LRRK2 conditional analyses. This independent locus is included for comparison to the LRRK2 variants because it is not expected to be affected by the complex linkage disequilibrium (LD) patterns within the LRRK2 region. Statistical power was calculated as described in the Supplementary Information.

### Co‐inheritance Analysis

We used two methods to compare the allelic distributions of the single nucleotide polymorphisms (SNPs) provided in Table 1 before and after removing the carriers of p.G2019S, rs76904798, and p.N2081D or a combination of these variants. First, we aggregated all samples used in the meta‐analysis and calculated the percentage of carriers of a particular SNP that were excluded in the conditioned data sets (Table S4). This was used as a disease‐independent measure of the co‐inheritance of the two variants. Second, we performed Fisher's exact test in R with Bonferroni correction to assess whether there were significant differences in the allelic distributions of each SNP between the unconditioned and conditioned data sets. For this comparison, only patients and proxy patients were included due to the large imbalance between the number of patients and controls in the UK Biobank. This analysis was performed separately for the IPDGC case, UK Biobank case, and proxy‐case data sets (Table S5).

**TABLE 1:** *LRRK2* variants examined in this study

MarkerName (hg19)	REF	ALT	RS‐ID	Region	Gene	Protein consequence	MAF unconditioned	MAF conditioned
12:40614434	C	T	rs76904798	Intergenic	LINC02471;LRRK2	NA	0.1451	0
12:40629436	T	C	rs33995463	Exonic	LRRK2	L119P	0.0026	0.0031
12:40657700	C	G	rs7308720	Exonic	LRRK2	N551K	0.0667	0.0767
12:40671989	A	G	rs10878307	Exonic	LRRK2	I723V	0.0684	0.0787
12:40702911	G	A	rs7133914	Exonic	LRRK2	R1398H	0.0692	0.0794
12:40707778	G	A	rs35507033	Exonic	LRRK2	R1514Q	0.0089	0.0026
12:40707861	C	T	rs33958906	Exonic	LRRK2	P1542S	0.029	0.0338
12:40713899	T	C	rs35303786	Exonic	LRRK2	M1646T	0.0169	0.0196
12:40713901	T	A	rs11564148	Exonic	LRRK2	S1647T	0.2979	0.3442
12:40734202	G	A	rs34637584	Exonic	LRRK2	G2019S	0.0009	0
12:40740686	A	G	rs33995883	Exonic	LRRK2	N2081D	0.0168	0.003
12:40758652^a^	C	T	rs3761863	Exonic	LRRK2	M2397T	0.3318	0.3536
12:123326598	G	T	rs10847864	Intronic	HIP1R	NA	0.3563	0.3558

All *LRRK2* missense variants with frequency >0.001 in the IPDGC, UK Biobank case–control and proxy‐control data sets, in addition to rs76904798, p.G2019S, and the independent chromosome 12 signal in HIP1R (rs10847864). MarkerName denotes the chromosome and base pair position with respect to reference assembly GRCh37/hg19. “REF” and “ALT” denote the reference and alternate alleles used in the association analysis. "RS‐ID" denotes the reference SNP cluster ID used by databases and researchers to refer to specific SNPs. “MAF unconditioned” and “MAF conditioned” denote the minor allele frequency from the combined IPDGC and UK Biobank data sets used in the unconditioned and conditioned (Δ p.G2019S Δ rs76904798) analyses, respectively. LRRK2, leucine‐rich repeat kinase 2; IPDGC, International Parkinson's Disease Genomics Consortium.

^a^ Association was based on the minor allele rs3761863‐T that corresponds to the protein consequence T2397M.

### Code Availability

All codes used in this study are available at https://github.com/neurogenetics/LRRK2_conditional_v3.

## Results

### Included Data Overview

We included 15 data sets, with 13 case–control cohorts (n_case = 16,309, n_control = 17,705), case–control data from the UK Biobank (n_case = 1529, n_control = 15,279), and proxy‐control data from the UK Biobank (n_proxy‐case = 13,404, n_control = 140,655). Together, these sample sets comprised 17,838 PD patients, 13,404 proxy patients, and 173,639 healthy controls of European ancestry. The mean age of onset for PD in the IPDGC cohorts, age at recruitment for the UK Biobank, and percentage of female participants for each data set are reported in Table S1, where this information was available.

### *LRRK2* rs76904798 Is Independently Associated with PD Risk From *LRRK2* Coding Variation

In the examination of the IPDGC, UK Biobank case–control, and proxy–control data sets, we identified 10 nonsynonymous *LRRK2* coding variants with a minor allele frequency (MAF) > 0.001 in all three data sets, as presented in Table 1. We verified the presence of these variants in the IPDGC data sets using whole‐genome sequencing data^28^ and in the UK Biobank data sets using whole‐exome sequencing data.^29^ The concordance rates were very high (average = 99.24%), showing that the imputation of these variants was highly accurate. The imputation quality and concordance values are presented in Tables S2 and S6, respectively.

Four of these variants (p.N551K, p.R1398H, p.M1646T, and p.N2081D) have been previously associated with either decreased or increased PD risk in Europeans,^7^, ^8^, ^30^ and the haplotype p.S1647T‐p.M2397T has been suggested to confer a protective effect in Taiwanese individuals.^31^ Although none of the four previously associated variants reached genome‐wide significance in our meta‐analysis (Table 2; Figs. S1–S4), the direction of the effects was consistent with previous studies.^7^, ^15^ We did not find any evidence of an association with PD for either p.S1647T or p.M2397T in this study (Figs. S5 and S6).

**TABLE 2:** Association results of *LRRK2* variants

RS‐ID	Protein consequence	Unconditioned, OR (95 CI)	Unconditioned, *P*‐value	Δ p.G2019S Δ rs76904798, OR (95 CI)	Δ p.G2019S Δ rs76904798, *P*‐value	Δ p.G2019S Δ p.N2081D, OR (95 CI)	Δ p.G2019S Δ p.N2081D, *P*‐value	HetISq	HetPVal
rs76904798	NA	1.12 (1.08–1.16)	4.01E‐09	NA	NA	1.11 (1.07–1.16)	1.40E‐07	23.793	0.04853
rs33995463	L119P	0.94 (0.69–1.29)	0.7223	0.91 (0.64–1.29)	0.5976	0.91 (0.66–1.25)	0.5521	5.687	0.7708
rs7308720	N551K	0.9 (0.85–0.95)	1.13E‐04	0.93 (0.88–0.99)	0.01506	0.91 (0.86–0.96)	4.55E‐04	8.957	0.8338
rs10878307	I723V	1 (0.95–1.06)	0.8648	1.03 (0.97–1.09)	0.3545	1.03 (0.98–1.08)	0.3042	23.402	0.05403
rs7133914	R1398H	0.9 (0.86–0.95)	1.31E‐04	0.93 (0.87–0.98)	0.01029	0.91 (0.86–0.96)	6.92E‐04	7.938	0.8925
rs35507033	R1514Q	1.13 (0.98–1.31)	0.1002	0.98 (0.66–1.47)	0.9402	1.14 (0.98–1.32)	0.08066	9.926	0.7676
rs33958906	P1542S	0.93 (0.86–1.01)	0.0807	0.97 (0.88–1.05)	0.4304	0.96 (0.88–1.04)	0.2771	17.997	0.2069
rs35303786	M1646T	1.18 (1.07–1.3)	9.15E‐04	1.16 (1.04–1.3)	6.59E‐03	1.19 (1.08–1.32)	6.32E‐04	21.122	0.09855
rs11564148	S1647T	0.99 (0.96–1.02)	0.4799	0.99 (0.96–1.03)	0.7366	0.98 (0.95–1.01)	0.1157	10.287	0.7409
rs34637584	G2019S	9.02 (6.14–13.25)	3.66E‐29	NA	NA	NA	NA	4.34	0.993
rs33995883	N2081D	1.18 (1.07–1.29)	6.59E‐04	1.2 (0.9–1.61)	0.2054	NA	NA	17.291	0.241
rs3761863	M2397T	1.01 (0.98–1.04)	0.5904	1.04 (1–1.07)	0.03988	1.01 (0.98–1.04)	0.7049	20.418	0.1175
rs10847864	NA	1.1 (1.07–1.13)	8.76E‐11	1.09 (1.06–1.13)	1.65E‐07	1.1 (1.06–1.13)	3.42E‐10	32.267	3.67E‐03

Odds ratios (OR), 95% confidence intervals (CI), and *P*‐values from the IPDGC and UK Biobank meta‐analyses are reported for all *LRRK2* variants examined in this study and the independent chromosome 12 signal in HIP1R (rs10847864). Heterozygosity estimates are based on a meta‐analysis of the unconditioned data sets. “HetISq” and “HetPVal” refer to the I^2^ and *P*‐value in the heterogeneity test, respectively. LRRK2, leucine‐rich repeat kinase 2; IPDGC, International Parkinson's Disease Genomics Consortium.

**FIG 1:**
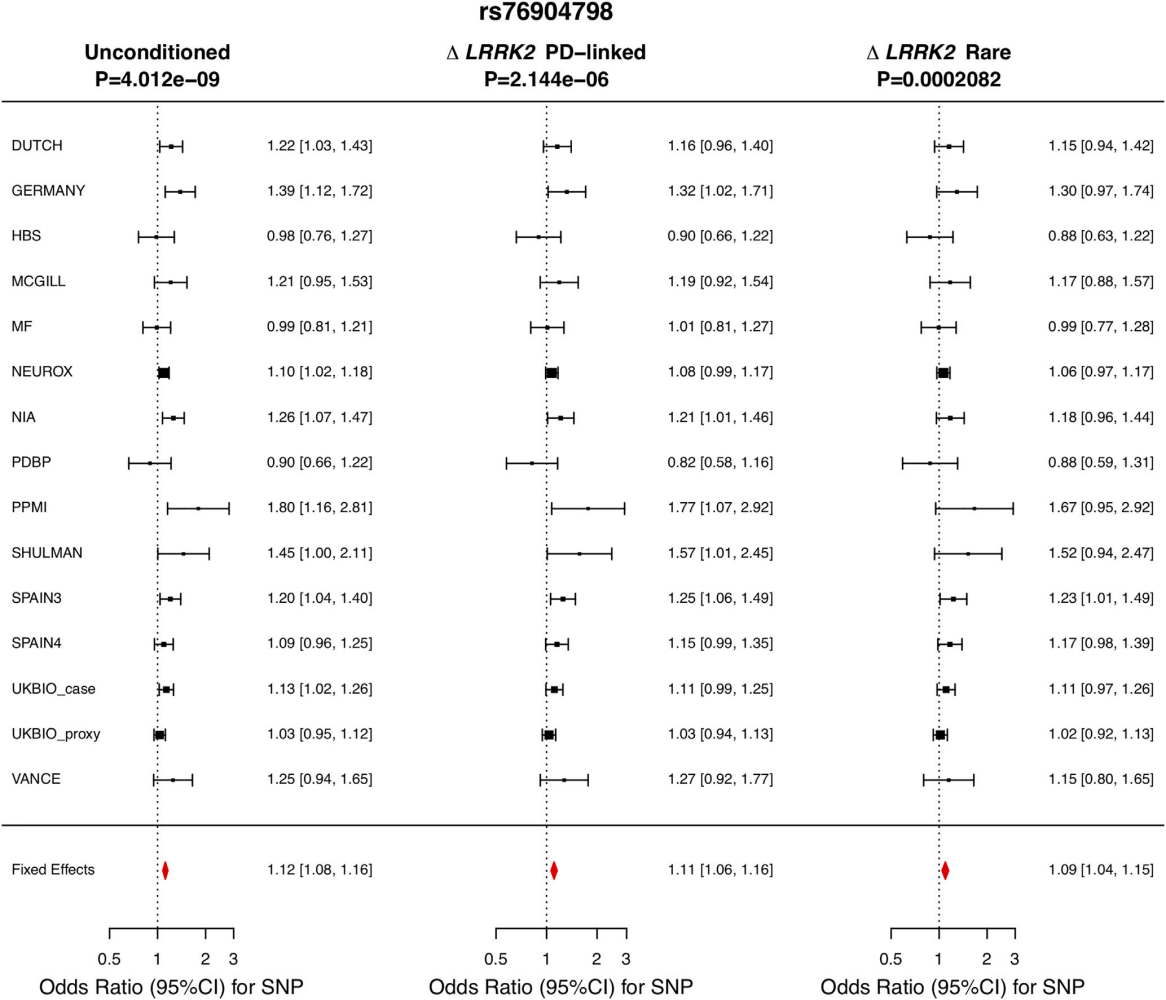
Meta‐analysis of rs76904798 in the included data sets excluding (from left to right) (1) no samples; (2) carriers of *LRRK2* p.G2019S, p.N551K, p.R1398H, p.M1646T, and p.N2081D; and (3) carriers of *LRRK2* p.G2019S, p.N551K, p.R1398H, p.M1646T, p.N2081D, p.L119P, p.I723V, p.R1514Q, and p.P1542S.

To confirm the effect of the previously identified PD GWAS loci in *LRRK2*, we first verified the associations of the known pathogenic variant p.G2019S (OR: 9.02, 95% CI: 6.14–13.25, *P* = 3.66E‐29) and the 5′ noncoding variant rs76904798 (OR: 1.12, 95% CI: 1.08–1.16, *P* = 4.01E‐09) in a meta‐analysis of all 15 data sets (Table 2; Fig. S7). We then confirmed the independent association of rs76904798 with PD risk from p.G2019S and p.N2081D (Δ p.G2019S Δ p.N2081D, OR: 1.11, 95% CI: 1.07–1.16, *P* = 1.40e−07) (Table 2; Fig. S8), the PD‐linked *LRRK2* coding variants (Δ p.G2019S Δ p.N551K Δ p.R1398H Δ p.M1646T Δ p.N2081D, OR: 1.11, 95% CI: 1.06–1.16, *P* = 2.144e−06), and all of the relatively rare *LRRK2* coding variants examined in this study (Δ p.G2019S Δ p.N551K Δ p.R1398H Δ p.M1646T Δ p.N2081D Δ p.L119P Δ p.I723V Δ p.R1514Q Δ p.P1542S, OR: 1.09, 95% CI: 1.04–1.15, *P* = 0.0002082) (Fig. 1). We used a Bonferroni‐corrected *P*‐value of 0.006 to determine the independence of rs76904798, which is based on the number of *LRRK2* coding variants excluding p.G2019S that were examined in this study (Table 1), two of which (p.N551K‐p.R1398H) represent the same allele (0.05/9 = 0.006). We also confirmed the independent association of rs76904798 by including the allele counts of the relatively rare *LRRK2* coding variants as covariates in a logistic regression model (OR: 1.11, 95% CI: 1.06–1.15, *P* = 9.90e‐07) (Table S7).

**FIG 2:**
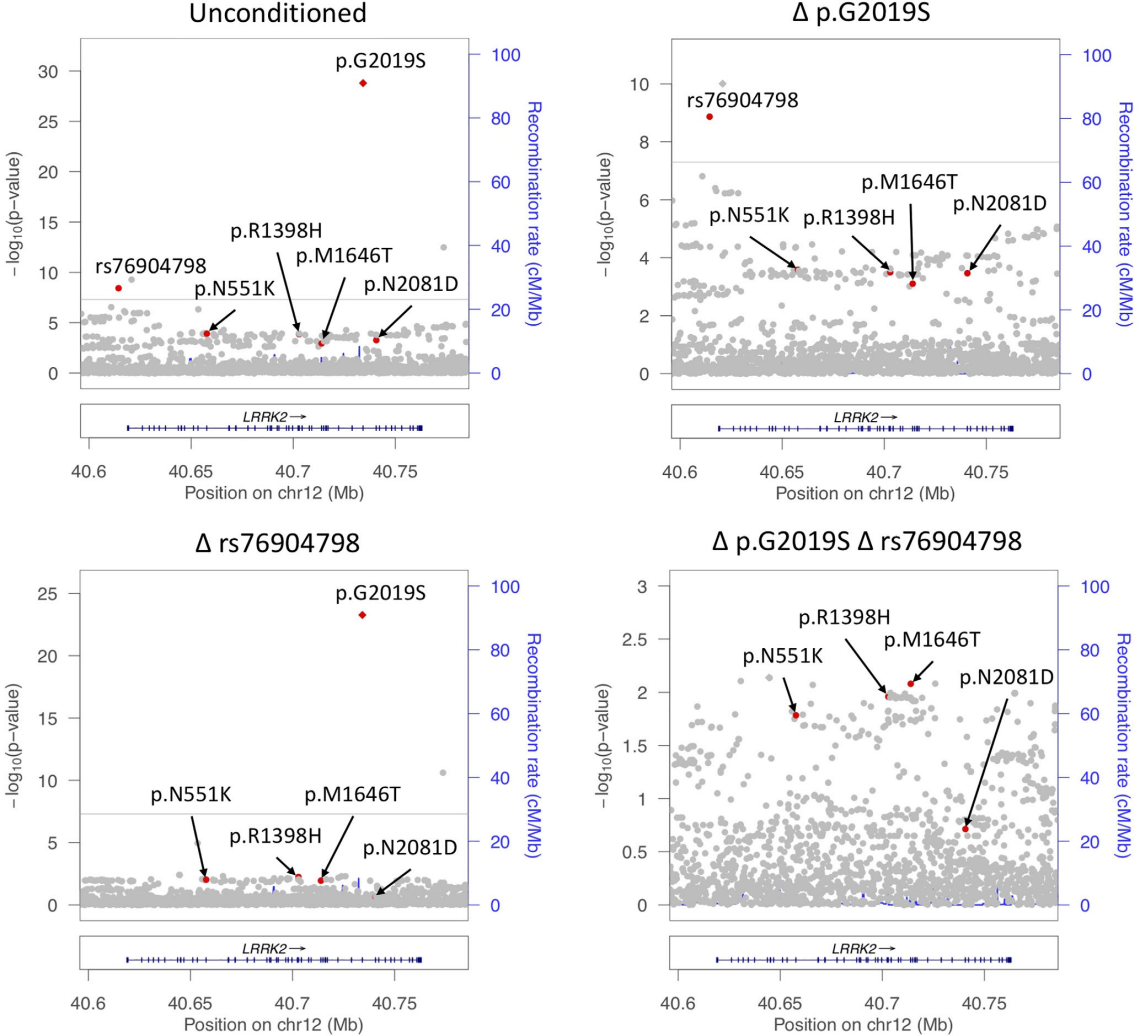
LocusZoom plot of *LRRK2* association with Parkinson's disease risk.

There were no other independent signals in the *LRRK2* region that reached genome‐wide significance (*P* < 5e‐8). The association of the 3′ intergenic variant rs190807041 disappears after conditioning on p.G2019S, and the 5′ signal driven by noncoding *LRRK2* variation explains the associations of both rs76904798 and the proximal intronic variant rs1491942 with PD risk (Fig. 2). The additional signal in the 5′ locus, rs1491942, is in complete LD with rs76904798, and this signal disappears when carriers of rs76904798 are removed.

### Conditional Analysis of *LRRK2* Exonic Variants Does Not Preclude an Independent Association of p.N551K‐p.R1398H and p.M1646T With Altered PD Risk

To better understand the role of coding variation at the *LRRK2* locus in PD, we performed a meta‐analysis excluding the carriers of p.G2019S and rs76904798, representing the two independent GWAS signals at the locus. In total, there were 12,504 PD patients (total removed: 29.9%, p.G2019S carriers: 1.4%, rs76904798 carriers: 28.8%), 9679 proxy patients (total removed: 27.8%, p.G2019S carriers: 0.2%, rs76904798 carriers: 27.6%), and 127,254 controls (total removed: 26.7%, p.G2019S carriers: 0.1%, rs76904798 carriers: 26.6%) in the conditional analysis. After removing carriers of p.G2019S and rs76904798, there were no *LRRK2* variants that reached genome‐wide significance. The results of the association analyses for the *LRRK2* variants examined in this study are presented in Table 2 and Table S3, and forest plots are presented in Figures S1–S6 and S8–S12. Forest plots of *LRRK2* p.K1423K, which is in high LD with p.N551K‐p.R1398H, and rs10847864 (*HIP1R* GWAS signal) are presented in Figures S13 and S14, respectively.

With this sample size, we had ~80% power to detect genotype relative risks ≥1.16 and ≥1.44 based on the MAFs of p.M1646T and p.N2081D, respectively, in the conditional analysis (Table 1). Similarly, we had ~80% power to detect genotype relative risks ≤0.93 based on the MAF of p.N551K and p.R1398H in the conditional analysis (Table 1). This calculation is based on the Bonferroni‐corrected significance threshold of *P* = 0.006.

None of the *LRRK2* variants examined in this study passed Bonferroni multiple test correction after excluding the carriers of p.G2019S and rs76904798, including the previously PD‐linked variants p.N551K (OR_condi: 0.93, 95% CI: 0.88–0.99, *P* = 0.01506), p.R1398H (OR_condi: 0.93, 95% CI: 0.87–0.98, *P* = 0.01029), p.M1646T (OR_condi: 1.16, 95% CI: 1.04–1.3, *P* = 0.00659), and p.N2081D (OR_condi: 1.2, 95% CI: 0.91–1.61, *P* = 0.2054) (Table 2; Figs. S1–S4). LocusZoom plots of the stepwise conditional analysis are shown in Figure 2. However, p.N551K, p.R1398H, and p.M1646T retained Bonferroni‐corrected significance when p.G2019S and rs76904798 allele counts were included as covariates in a logistic regression model, though they still did not meet genome‐wide significance (Table S7). We also performed a meta‐analysis excluding the carriers of p.N2081D and p.G2019S because rs76904798 is often co‐inherited with p.N2081D (Fig. S15). After only the p.G2019S carriers were removed, *LRRK2* p.N551K, p.R1398H, p.M1646T, and p.N2081D passed Bonferroni multiple test correction (Table S3).

### Rare *LRRK2* Missense Variants Are Co‐Inherited With rs76904798

To further investigate the effects of excluding rs76904798 carriers on the allele distribution of *LRRK2* coding variants, we calculated the percentage of carriers that were removed after excluding the carriers of p.G2019S and rs76904798 in our data sets, as shown in Table S4. We found that 86.64% of p.N2081D carriers and 79.07% of p.R1514Q carriers were excluded after removing the carriers of rs76904798 from all 15 data sets. Only 0.19% of p.N2081D carriers and 0.08% of p.R1514Q carriers were excluded after removing p.G2019S carriers, all of which also carried rs76904798. Conversely, only 10.72% of rs76904798 carriers were excluded after removing p.N2081D carriers. Because the 5′ variant rs76904798 remained associated with PD after removing the carriers of p.G2019S and p.N2081D (Fig. S8), the GWAS signal represented by rs76904798 is independent of p.N2081D, and the previous significance reported for p.N2081D might be due to the rs76904798 signal.

We also found significant differences in the allele distributions of p.R1514Q, p.N2081D, and p.S1647T after removing rs76904798 carriers from the IPDGC case, UK Biobank case, and proxy‐case data sets (Table S5). Although p.S1647T was significantly enriched among noncarriers of rs76904798, this variant was not associated with PD, and around 17% of carriers were excluded after removing rs76904798 carriers. Only around 16% of p.N551K and p.R1398H carriers were excluded after removing rs76904798 carriers, and less than 1% after removing p.G2019S carriers. The complete results of the co‐inheritance analysis are presented in Tables S4 and S5, and the frequencies and allele distributions of the *LRRK2* variants examined in this study are presented in Table S8.

## Discussion

It has been established that both rare and common variations at the *LRRK2* locus can influence PD susceptibility. Several large GWASs and meta‐analyses have pointed to at least two independent association signals, represented by p.G2019S and 5′ noncoding variation, that reach genome‐wide significance.^15^ However, the literature has often produced conflicting or inconclusive results when it comes to PD risk associated with common variation at the *LRRK2* locus. Interestingly, several *LRRK2* variants have been associated with other disorders, suggesting that there are shared pathological mechanisms that result from genetic changes in *LRRK2*. For example, *LRRK2* p.N2081D has been implicated as a shared risk variant in Crohn's disease,^8^ and independent *LRRK2* 5' variation has been shown to be a modifier of progressive supranuclear palsy phenotypes.^32^ To refine our understanding of the pattern of *LRRK2* association with PD, we investigated the PD risk associated with *LRRK2* missense variants in a large meta‐analysis of European‐ancestry individuals.

First, we confirmed the genome‐wide associations of p.G2019S and rs76904798 and identified the potential associations of four other PD‐linked *LRRK2* missense variants (p.N551K, p.R1398H, p.M1646T, and p.N2081D), all of which passed multiple test correction. After removing carriers of the common noncoding variant rs76904798, we found that these missense variants do not pass Bonferroni correction, whereas they retained Bonferroni‐corrected significance when rs76904798 was included as a covariate in a logistic regression model. These data therefore do not exclude the possibility that the putative protective haplotype p.N551K‐p.R1398H and proposed risk factor p.M1646T are independently associated with PD from rs76904798. These variants, however, did not meet genome‐wide significance in our study despite including a very large sample size.

In a recent analysis of whole‐genome sequencing data from the Accelerating Medicines Partnership‐Parkinson's Disease cohort, Bryant and colleagues found that the 5′ variant rs76904798 was associated with *LRRK2* p.R1514Q and p.N2081D.^33^ We confirmed the co‐inheritance of these variants with rs76904798 in a data set that is ~20 times larger and also found a significant difference in the allele distribution of p.S1647T after removing rs76904798 carriers. Our results suggest that any association between p.N2081D and PD is very likely due to linkage disequilibrium with rs76904798. Bryant and colleagues suggested that p.N2081D may be important in mediating disease risk associated with the 5′ noncoding variation. However, we establish here that rs76904798 is independently associated with PD from p.N2081D, and therefore, the role of p.N2081D in PD pathogenesis needs to be explored further and potentially reclassified.

Several *LRRK2* variants have been implicated in mechanisms that are relevant to PD pathogenesis. LRRK2 rs76904798 has been previously associated with increased LRRK2 expression in monocytes 19 and in human brain and iPSC‐derived microglia 21. To further investigate the other potential effects of this variant on expression, we queried the Open Targets Platform (v. 21.06) in July 2021 for eQTL data across tissue and cell types. LRRK2 expression linked to this variant was significant across multiple tissues, including artery, skin, and thyroid, as well as monocyte cells, which is concordant with previously cited literature. The most significant expression signal was from whole‐blood eQTL data sourced from eQTLgen (beta = 0.290, *P* = 1.3e‐111). Increased LRRK2 expression in whole blood and monocytes is supportive of previous studies that suggest LRRK2 has a role in immune regulation and inflammation.^34^, ^35^ Based on public data sources and previous literature, rs76904798 has significant evidence for increasing LRRK2 expression in immune‐related cells.

Previous studies of the p.G2019S mutation have shown that increased *LRRK2* kinase activity, typically estimated to be approximately twofold compared to wild type, has negative effects on neuronal survival.^36^, ^37^, ^38^, ^39^ Both p.N2081D and p.M1646T are also associated with increased kinase activity compared to wild‐type *LRRK2*,^8^, ^40^ although the effect size is modest compared to p.G2019S. Despite this evidence, it is possible that prior data using transfection of plasmids in HEK293T cells are unable to disambiguate the effects of p.N2081D and rs76904798 as they are often present on the same haplotype. *LRRK2* p.M1646T has also been associated with increased glucocerebrosidase activity with a larger effect than p.G2019S.^30^ In addition, *LRRK2* p.R1398H has been shown to affect GTPase and Wnt signaling activity contrary to pathogenic *LRRK2* mutations.^8^, ^41^, ^42^, ^43^ A recent study has shown that both p.R1398H and p.N551K were able to counteract the putative pathogenic effects of p.G2019S in *Drosophila* models.^44^ The literature therefore suggests that each of these variants has measurable effects on protein function in cells and in vivo that are consistent with the proposed direction of effects for risk versus protection. However, the relation between the functional impact and risk for PD needs to be further investigated for these coding variants.

Previous studies have also implicated the common *LRRK2* variant p.M2397T in several disorders. Although this variant does not appear to affect kinase activity,^38^ the allele M2397 has been reported to lower LRRK2 abundance due to protein destabilization,^45^ which has been related to enhanced inflammatory responses in both Crohn's disease^45^, ^46^ and leprosy.^47^, ^48^ Despite the fact that p.M2397T has demonstrated a protective association with PD in a study consisting of 573 Taiwanese patients,^31^ and with MSA—a disease that shares many clinical and pathological features with PD—in a study consisting of 177 neuropathologically confirmed patients from America and the United Kingdom,^49^ we did not find any evidence that p.M2397T was associated with PD in this study. It is possible that previous studies linking *LRRK2* p.M2397T with PD and MSA were underpowered due to relatively small sample sizes, and further investigation in larger and diverse cohorts is warranted.

We note several limitations of this study. First, our use of imputed genotype data limits the accuracy of some genotypes, though very high concordance of the *LRRK2* coding variants with whole‐genome and whole‐exome sequence data shows that the imputation of these variants was highly accurate. Second, although we included a large amount of data in our meta‐analysis, removing over one‐quarter of the samples in the conditional analysis limited our statistical power to detect less‐common small‐effect (OR ~ 1.2) variants. Therefore, we cannot exclude that relatively common coding variants have a very small effect resulting in increased or decreased risk for PD. Further studies exploring the cumulative effects of common small‐effect variants could enhance our understanding of their genetic burden and should consider the complex linkage disequilibrium structure of the *LRRK2* region. Third, our analysis was limited to individuals of European ancestry, so the results of this study may not extend beyond European‐ancestry populations. In‐depth studies of PD‐linked genes in diverse cohorts will help clarify the associations discussed here as well as broader disease mechanisms.

In conclusion, here we provide insights into the relationship between coding and noncoding variation at the *LRRK2* locus and demonstrate that the 5′ *LRRK2* noncoding GWAS signal represented by rs76904798 is independently associated with PD risk from *LRRK2* coding variation. These coding variants are therefore unlikely to drive the *LRRK2* GWAS signal in individuals of European ancestry and require additional genetic and functional studies to clarify their potential impact on disease state. Characterizing the PD susceptibility associated with *LRRK2* genetic variation is critical for anticipating disease progression and response to *LRRK2*‐targeted therapeutics.

## AuthorRoles

Concept and design: C.B., H.L.L.

Statistical analysis: J.L., M.A.N., C.B., H.L.L.

Contributed expertise, data, or DNA samples: X.R., R.L., M.R.C., Z.G.‐O.

Drafting of the manuscript: All authors

Critical revision of the manuscript: All authors

## Supporting information


**APPENDIX S1** Supporting Information

**Figure S1.** Meta‐analysis of p.N551K in the included data sets excluding (from left to right) (1) no samples, (2) carriers of rs76904798 and p.G2019S, and (3) carriers of p.N2081D and p.G2019S.
**Figure S2.** Meta‐analysis of p.R1398H in the included data sets excluding (from left to right) (1) no samples, (2) carriers of rs76904798 and p.G2019S, and (3) carriers of p.N2081D and p.G2019S.
**Figure S3.** Meta‐analysis of p.M1646T in the included data sets excluding (from left to right) (1) no samples, (2) carriers of rs76904798 and p.G2019S, and (3) carriers of p.N2081D and p.G2019S.
**Figure S4.** Meta‐analysis of p.N2081D in the included data sets excluding (from left to right) (1) no samples and (2) carriers of rs76904798 and p.G2019S.
**Figure S5.** Meta‐analysis of p.S1647T in the included data sets excluding (from left to right) (1) no samples, (2) carriers of rs76904798 and p.G2019S, and (3) carriers of p.N2081D and p.G2019S.
**Figure S6.** Meta‐analysis of p.M2397T in the included data sets excluding (from left to right) (1) no samples, (2) carriers of rs76904798 and p.G2019S, and (3) carriers of p.N2081D and p.G2019S.
**Figure S7.** Meta‐analysis of (from left to right) (1) p.G2019S and (2) rs76904798 in the included data sets.
**Figure S8.** Meta‐analysis of rs76904798 in the included data sets excluding (from left to right) (1) no samples and (2) carriers of p.N2081D and p.G2019S.
**Figure S9.** Meta‐analysis of p.L119P in the included data sets excluding (from left to right) (1) no samples, (2) carriers of rs76904798 and p.G2019S, and (3) carriers of p.N2081D and p.G2019S.
**Figure S10.** Meta‐analysis of p.I723V in the included data sets excluding (from left to right) (1) no samples, (2) carriers of rs76904798 and p.G2019S, and (3) carriers of p.N2081D and p.G2019S.
**Figure S11.** Meta‐analysis of p.R1514Q in the included data sets excluding (from left to right) (1) no samples, (2) carriers of rs76904798 and p.G2019S, and (3) carriers of p.N2081D and p.G2019S.
**Figure S12.** Meta‐analysis of p.P1542S in the included data sets excluding (from left to right) (1) no samples, (2) carriers of rs76904798 and p.G2019S, and (3) carriers of p.N2081D and p.G2019S.
**Figure S13.** Meta‐analysis of p.K1423K in the included data sets excluding (from left to right) (1) no samples, (2) carriers of rs76904798 and p.G2019S, and (3) carriers of p.N2081D and p.G2019S.
**Figure S14.** Meta‐analysis of rs10847864 in the included data sets excluding (from left to right) (1) no samples, (2) carriers of rs76904798 and p.G2019S, and (3) carriers of p.N2081D and p.G2019S.
**Figure S15.** LocusZoom plot of *LRRK2* association with Parkinson's disease risk conditioned on p.N2081D. The left panel shows the association signal at the *LRRK2* locus in the IPDGC and UK Biobank meta‐analysis conditioned on p.N2081D, and the right panel conditions on both p.G2019S and p.N2081D. The LRRK2 variants p.N551K, p.R1398H, p.M1646T, p.G2019S, and rs76904798 are indicated by red dots.

**Table S1.** Overview of included data.
**Table S2.** Imputation quality of LRRK2 variants examined in this study.
**Table S3.** Summary statistics of LRRK2 variants after removing carriers of the specified variants.
**Table S4.** Co‐inheritance analysis of LRRK2 variants examined in this study with p.G2019S, rs76904798, and p.N2081D.
**Table S5.** Fisher's exact test comparing the allele distributions of LRRK2 variants among cases in the unconditioned and conditioned data sets.
**Table S6.** Concordance of LRRK2 variants in the imputed data sets with whole‐exome sequencing data (IPDGC) and whole‐genome sequencing data (UKBIO).
**Table S7.** Summary statistics of LRRK2 variants, including the allele counts of the specified variants as covariates.
**Table S8.** Frequencies and allele distributions of LRRK2 variants examined in this study in the unconditioned and conditioned data sets.

## Data Availability

UK Biobank data are available via application (https://www.ukbiobank.ac.uk/). Various GWAS summary statistics from the IPDGC data are publicly available (https://pdgenetics.org/resources).
