# Supplementary material for: Coding and Noncoding Variation in LRRK2 and Parkinson's Disease Risk
Source: Mov Disord. 2021 Sep 20;37(1):95–105. doi: 10.1002/mds.28787 (PMC9292230; doi:10.1002/mds.28787)
Supplement: Supplementary file 2 — Figure S1. Meta‐analysis of p.N551K in the included data sets excluding (from left to right) (1) no samples, (2) carriers of rs76904798 and p.G2019S, and (3) carriers of p.N2081D and p.G2019S. Figure S2. Meta‐analysis of p.R1398H in the included data sets excluding (from left to right) (1) no samples, (2) carriers of rs76904798 and p.G2019S, and (3) carriers of p.N2081D and p.G2019S. Figure S3. Meta‐analysis of p.M1646T in the included data sets excluding (from left to right) (1) no samples, (2) carriers of rs76904798 and p.G2019S, and (3) carriers of p.N2081D and p.G2019S. Figure S4. Meta‐analysis of p.N2081D in the included data sets excluding (from left to right) (1) no samples and (2) carriers of rs76904798 and p.G2019S. Figure S5. Meta‐analysis of p.S1647T in the included data sets excluding (from left to right) (1) no samples, (2) carriers of rs76904798 and p.G2019S, and (3) carriers of p.N2081D and p.G2019S. Figure S6. Meta‐analysis of p.M2397T in the included data sets excluding (from left to right) (1) no samples, (2) carriers of rs76904798 and p.G2019S, and (3) carriers of p.N2081D and p.G2019S. Figure S7. Meta‐analysis of (from left to right) (1) p.G2019S and (2) rs76904798 in the included data sets. Figure S8. Meta‐analysis of rs76904798 in the included data sets excluding (from left to right) (1) no samples and (2) carriers of p.N2081D and p.G2019S. Figure S9. Meta‐analysis of p.L119P in the included data sets excluding (from left to right) (1) no samples, (2) carriers of rs76904798 and p.G2019S, and (3) carriers of p.N2081D and p.G2019S. Figure S10. Meta‐analysis of p.I723V in the included data sets excluding (from left to right) (1) no samples, (2) carriers of rs76904798 and p.G2019S, and (3) carriers of p.N2081D and p.G2019S. Figure S11. Meta‐analysis of p.R1514Q in the included data sets excluding (from left to right) (1) no samples, (2) carriers of rs76904798 and p.G2019S, and (3) carriers of p.N2081D and p.G2019S. Figure S12. Meta‐analysis of p.P1 [file MDS-37-95-s001.docx]

**Coding and non-coding variation in *LRRK2* and Parkinson's Disease Risk**

**Supplementary Figure 1.** Meta-analysis of p.N551K in the included datasets excluding (from left to right) 1) no samples 2) carriers of rs76904798 and p.G2019S and 3) carriers of p.N2081D and p.G2019S.

**Supplementary Figure 2.** Meta-analysis of p.R1398H in the included datasets excluding (from left to right) 1) no samples 2) carriers of rs76904798 and p.G2019S and 3) carriers of p.N2081D and p.G2019S.

**Supplementary Figure 3.** Meta-analysis of p.M1646T in the included datasets excluding (from left to right) 1) no samples 2) carriers of rs76904798 and p.G2019S and 3) carriers of p.N2081D and p.G2019S.

**Supplementary Figure 4.** Meta-analysis of p.N2081D in the included datasets excluding (from left to right) 1) no samples and 2) carriers of rs76904798 and p.G2019S.

**Supplementary Figure 5.** Meta-analysis of p.S1647T in the included datasets excluding (from left to right) 1) no samples 2) carriers of rs76904798 and p.G2019S and 3) carriers of p.N2081D and p.G2019S.

**Supplementary Figure 6.** Meta-analysis of p.M2397T in the included datasets excluding (from left to right) 1) no samples 2) carriers of rs76904798 and p.G2019S and 3) carriers of p.N2081D and p.G2019S.

**Supplementary Figure 7.** Meta-analysis of (from left to right) 1) p.G2019S and 2) rs76904798 in the included datasets.

**Supplementary Figure 8.** Meta-analysis of rs76904798 in the included datasets excluding (from left to right) 1) no samples and 2) carriers of p.N2081D and p.G2019S.

**Supplementary Figure 9.** Meta-analysis of p.L119P in the included datasets excluding (from left to right) 1) no samples 2) carriers of rs76904798 and p.G2019S and 3) carriers of p.N2081D and p.G2019S.

**Supplementary Figure 10.** Meta-analysis of p.I723V in the included datasets excluding (from left to right) 1) no samples 2) carriers of rs76904798 and p.G2019S and 3) carriers of p.N2081D and p.G2019S.

**Supplementary Figure 11.** Meta-analysis of p.R1514Q in the included datasets excluding (from left to right) 1) no samples 2) carriers of rs76904798 and p.G2019S and 3) carriers of p.N2081D and p.G2019S.

**Supplementary Figure 12.** Meta-analysis of p.P1542S in the included datasets excluding (from left to right) 1) no samples 2) carriers of rs76904798 and p.G2019S and 3) carriers of p.N2081D and p.G2019S.

**Supplementary Figure 13.** Meta-analysis of p.K1423K in the included datasets excluding (from left to right) 1) no samples 2) carriers of rs76904798 and p.G2019S and 3) carriers of p.N2081D and p.G2019S.

**Supplementary Figure 14.** Meta-analysis of rs10847864 in the included datasets excluding (from left to right) 1) no samples 2) carriers of rs76904798 and p.G2019S and 3) carriers of p.N2081D and p.G2019S.

**Supplementary Figure 15.** LocusZoom plot of *LRRK2* association with Parkinson’s disease risk conditioned on p.N2081D. The left panel shows the association signal at the *LRRK2* locus in the IPDGC and UK Biobank meta-analysis conditioned on p.N2081D, and the right panel conditions on both p.G2019S and p.N2081D. The LRRK2 variants p.N551K, p.R1398H, p.M1646T, p.G2019S and rs76904798 are indicated by red dots.


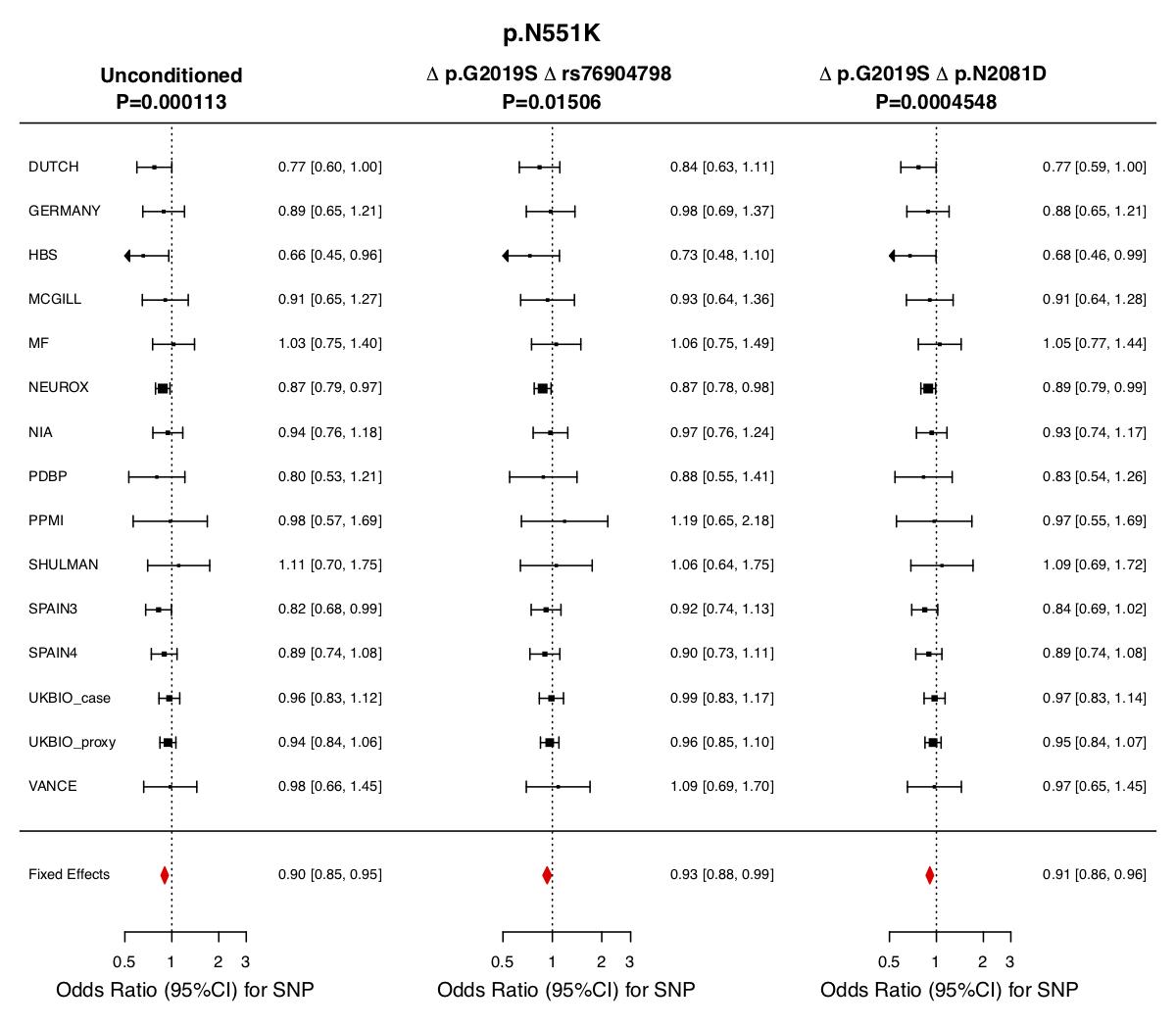


**Supplementary Figure 1.** Meta-analysis of p.N551K in the included datasets excluding (from left to right) 1) no samples 2) carriers of rs76904798 and p.G2019S and 3) carriers of p.N2081D and p.G2019S.


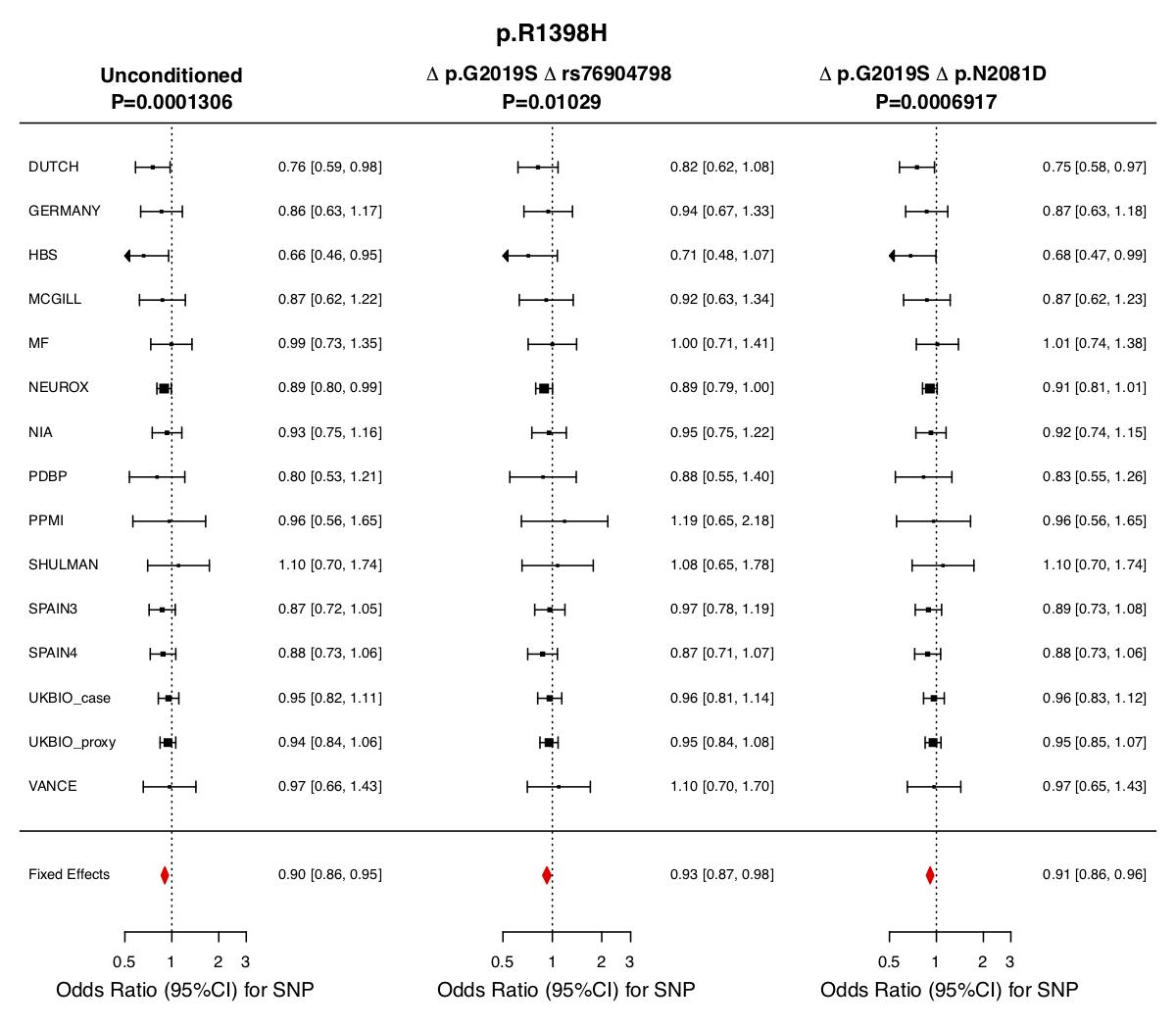


**Supplementary Figure 2.** Meta-analysis of p.R1398H in the included datasets excluding (from left to right) 1) no samples 2) carriers of rs76904798 and p.G2019S and 3) carriers of p.N2081D and p.G2019S.


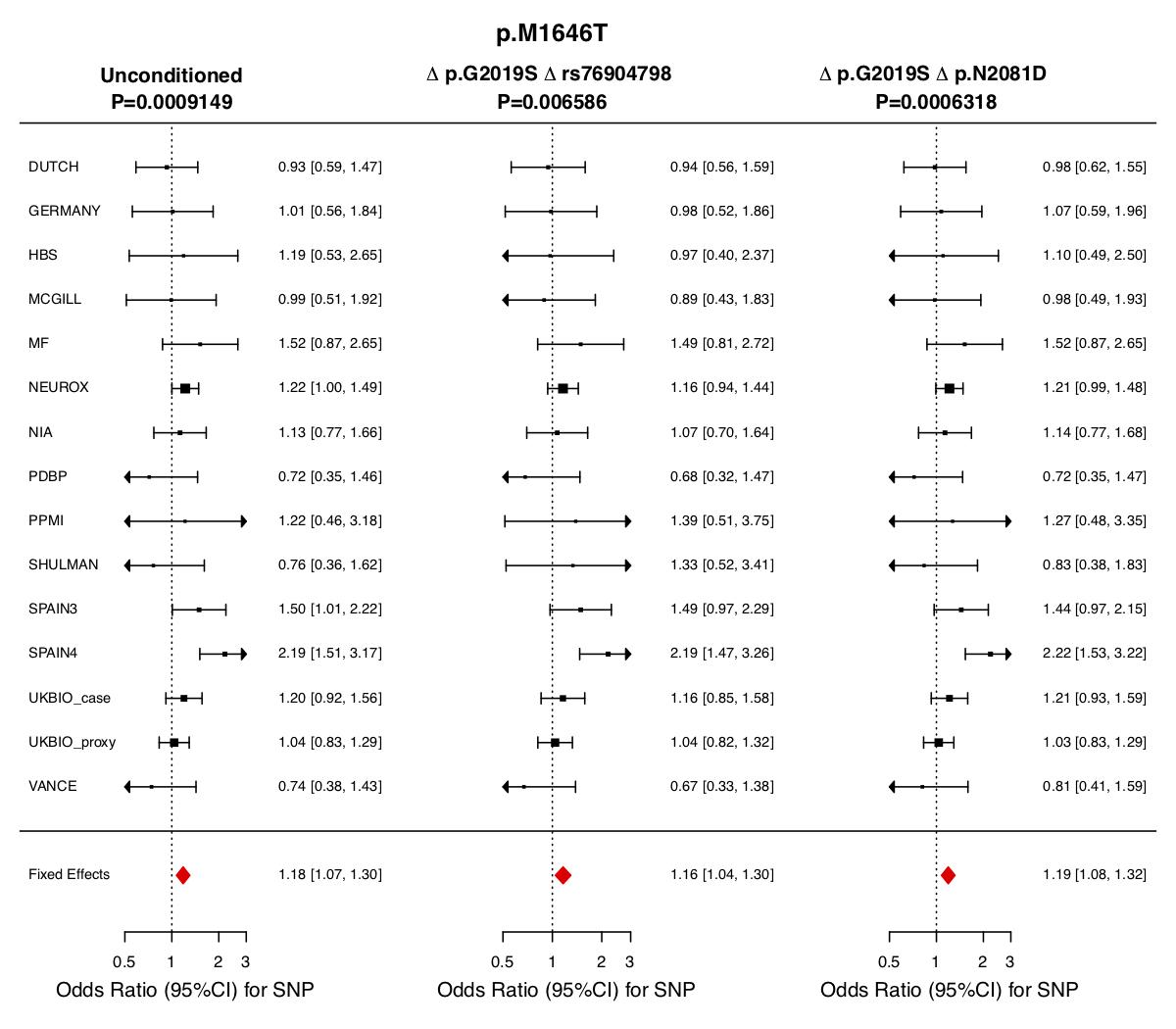


**Supplementary Figure 3.** Meta-analysis of p.M1646T in the included datasets excluding (from left to right) 1) no samples 2) carriers of rs76904798 and p.G2019S and 3) carriers of p.N2081D and p.G2019S.


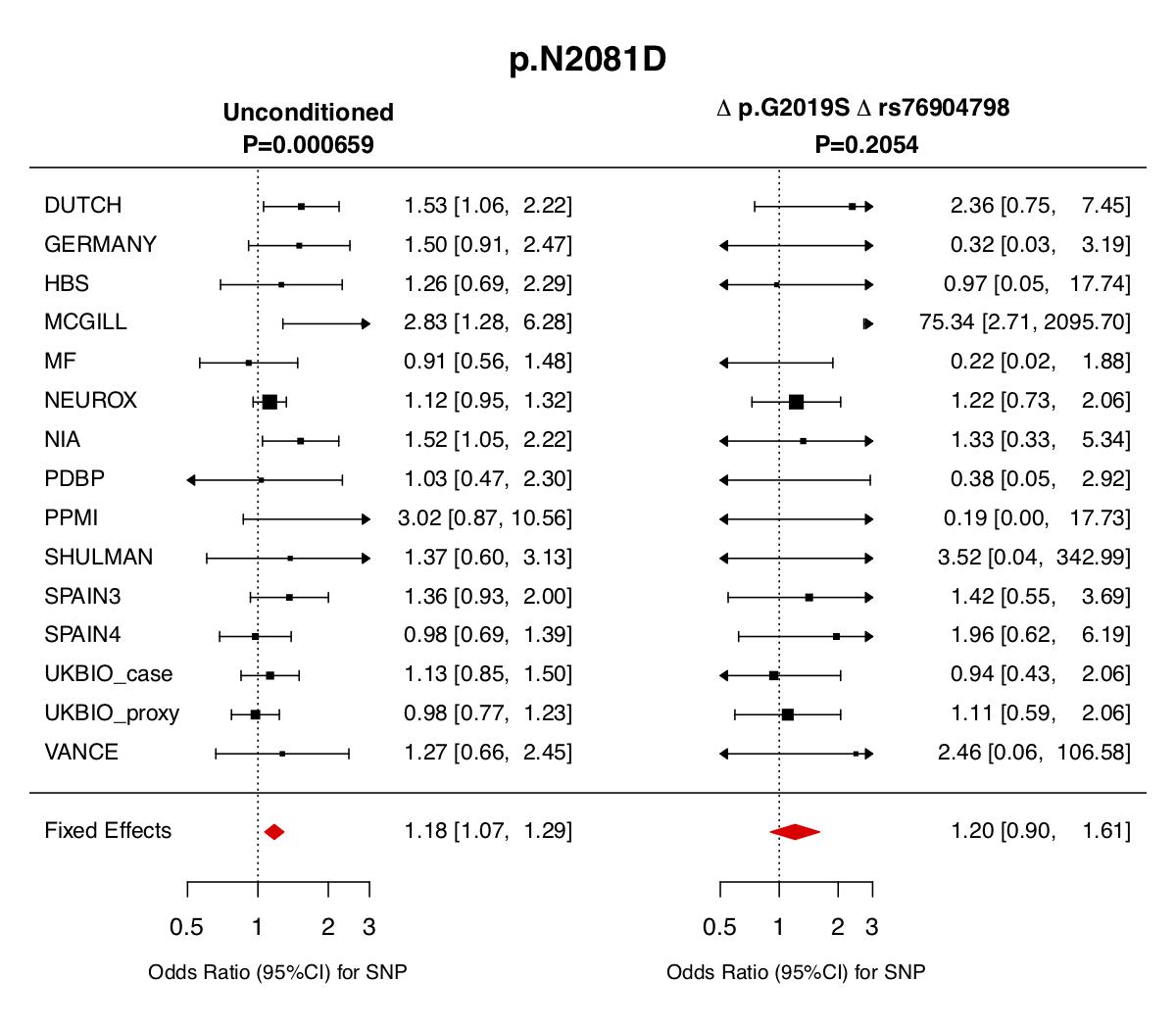


**Supplementary Figure 4.** Meta-analysis of p.N2081D in the included datasets excluding (from left to right) 1) no samples and 2) carriers of rs76904798 and p.G2019S.


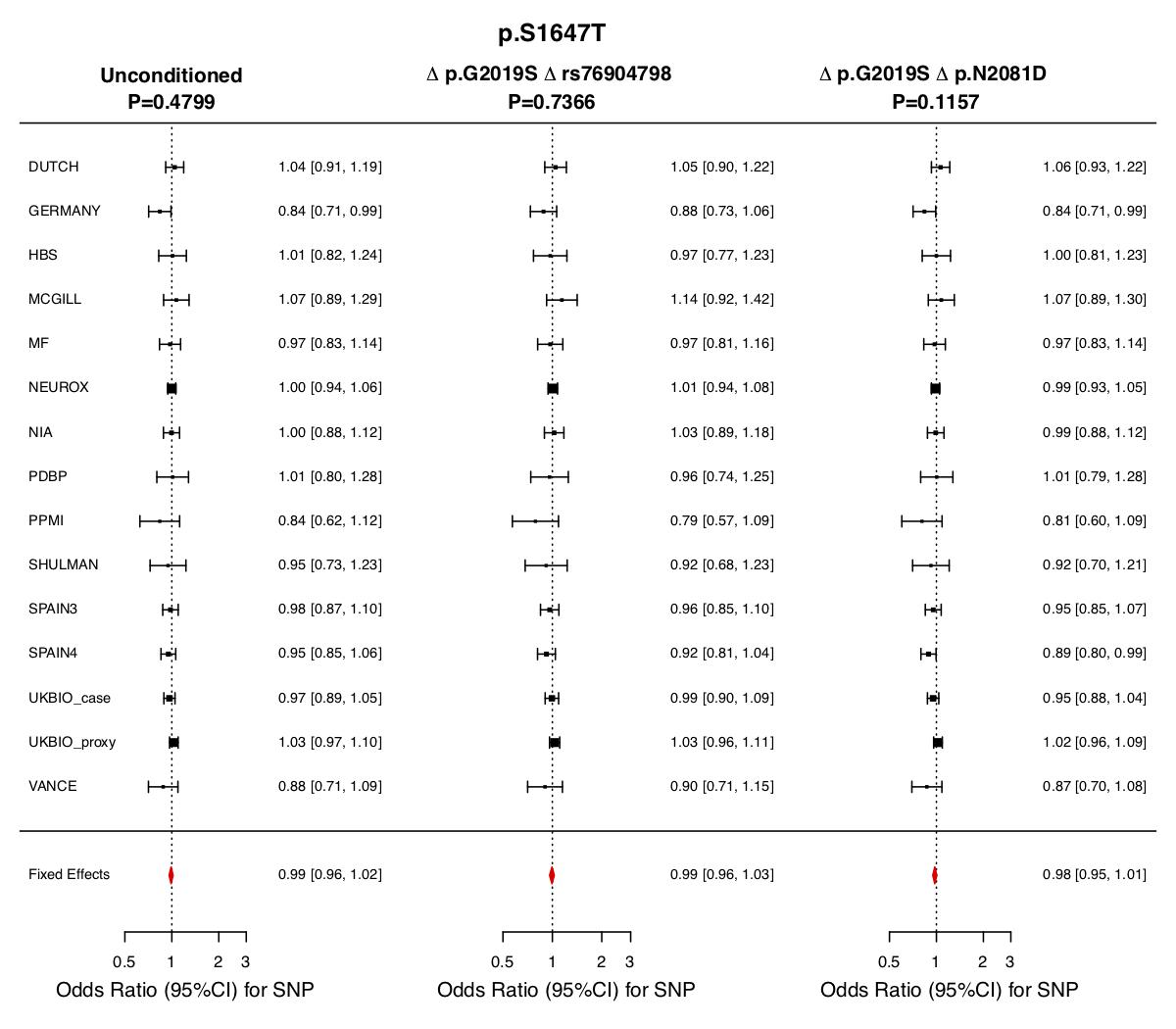


**Supplementary Figure 5.** Meta-analysis of p.S1647T in the included datasets excluding (from left to right) 1) no samples 2) carriers of rs76904798 and p.G2019S and 3) carriers of p.N2081D and p.G2019S.

**
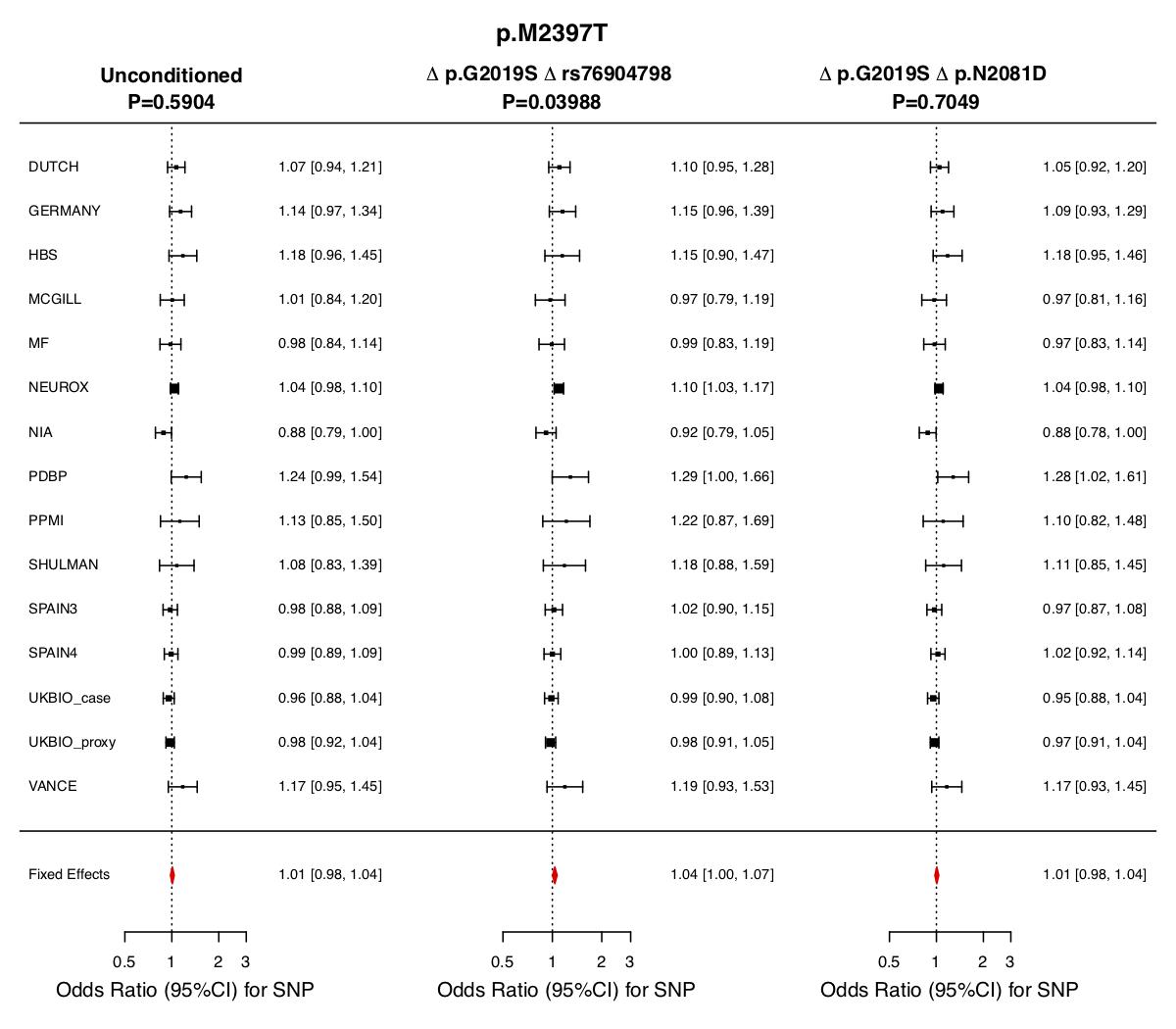
Supplementary Figure 6.** Meta-analysis of p.M2397T in the included datasets excluding (from left to right) 1) no samples 2) carriers of rs76904798 and p.G2019S and 3) carriers of p.N2081D and p.G2019S.

**
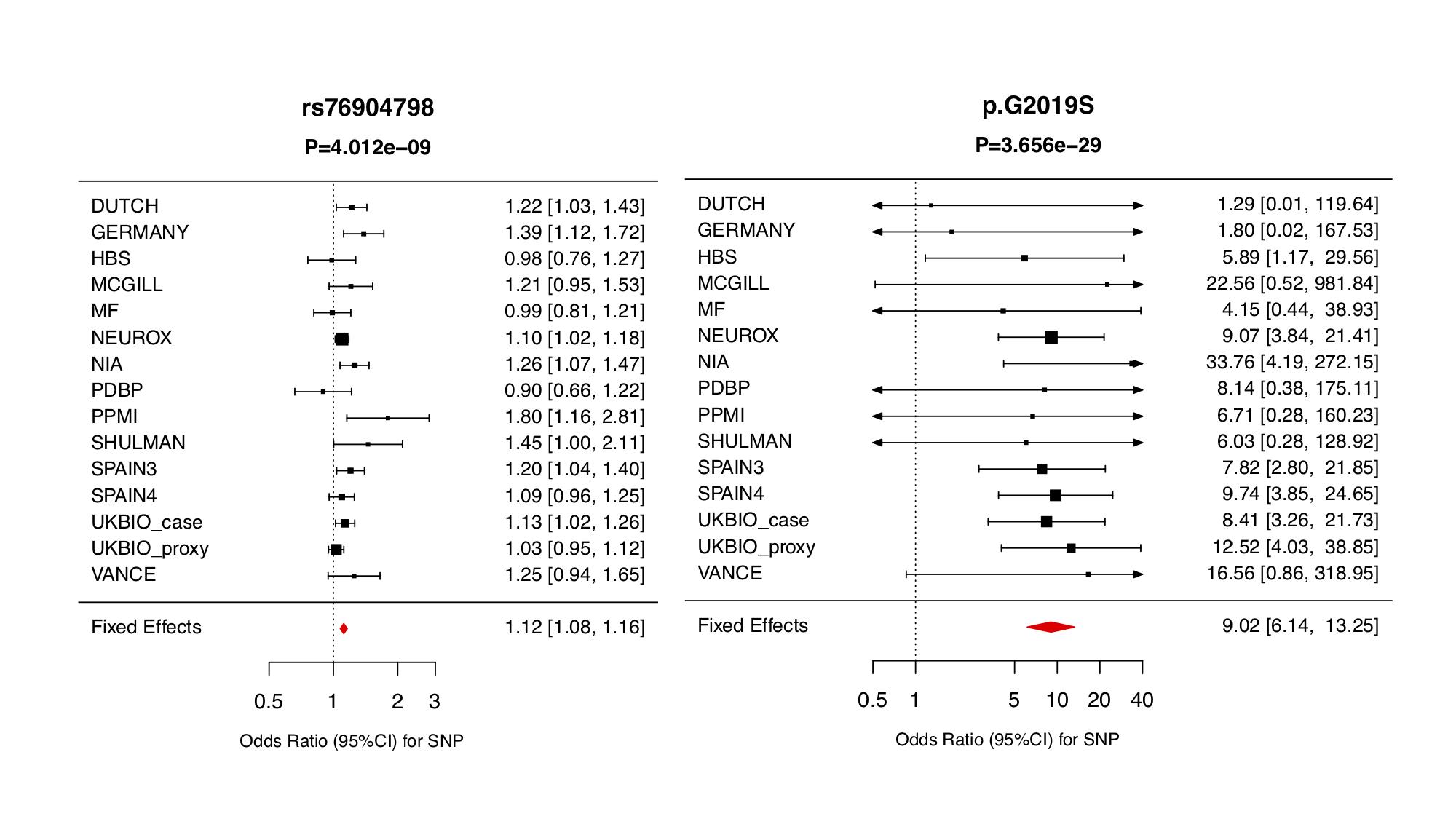
**

**Supplementary Figure 7.** Meta-analysis of (from left to right) 1) rs76904798 and 2) p.G2019S in the included datasets.


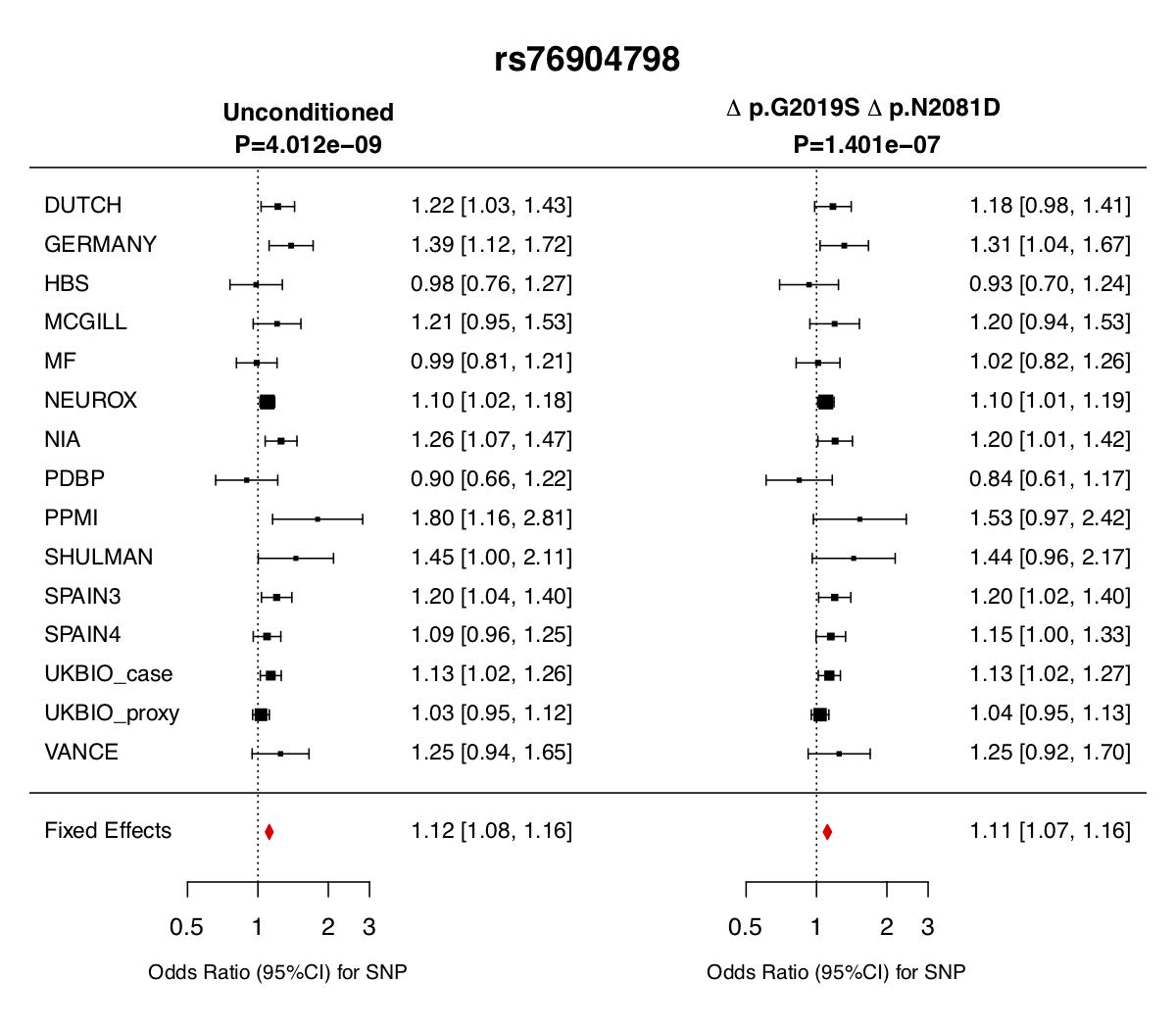


**Supplementary Figure 8.** Meta-analysis of rs76904798 in the included datasets excluding (from left to right) 1) no samples and 2) carriers of p.N2081D and p.G2019S.


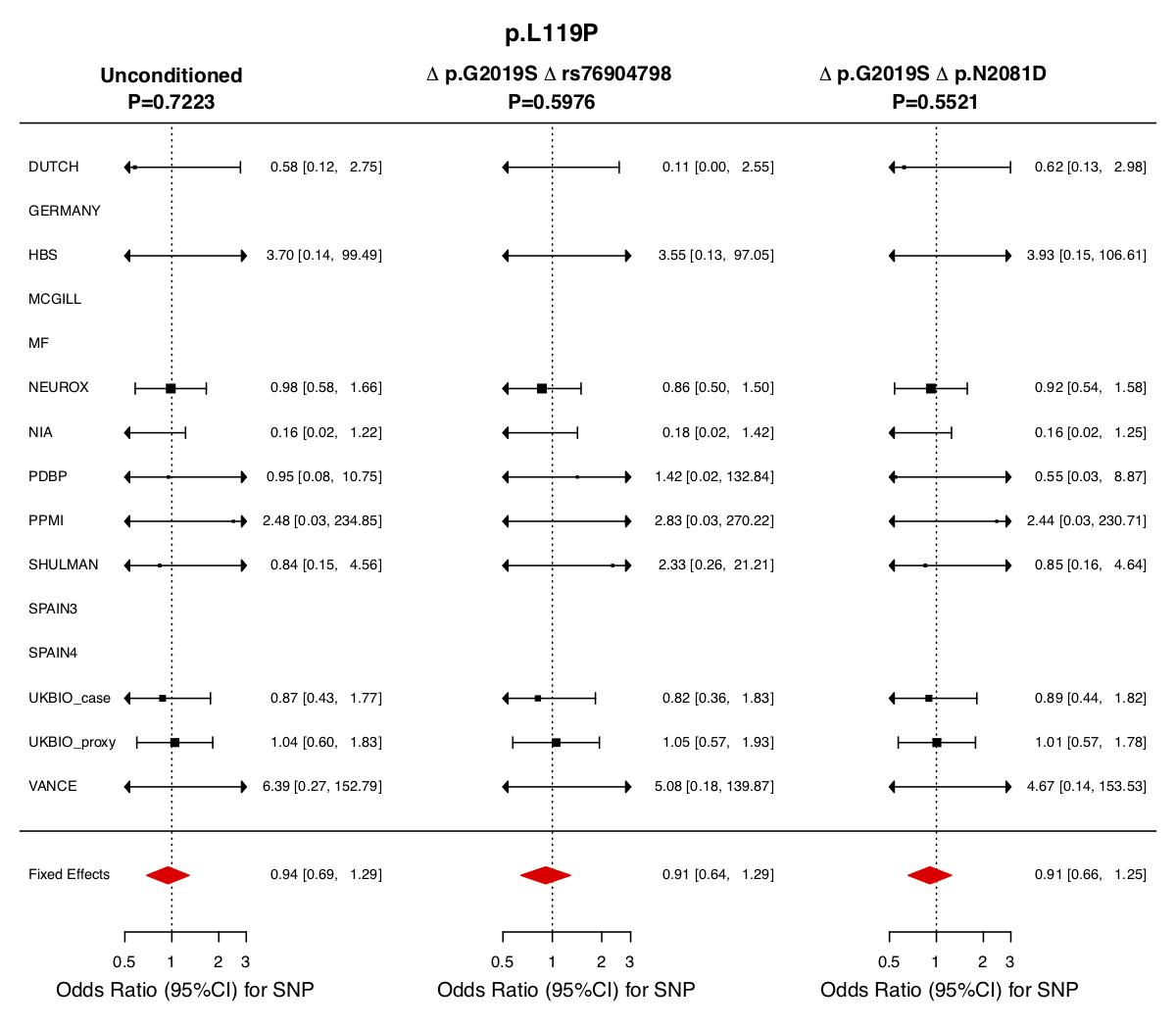


**Supplementary Figure 9.** Meta-analysis of p.L119P in the included datasets excluding (from left to right) 1) no samples 2) carriers of rs76904798 and p.G2019S and 3) carriers of p.N2081D and p.G2019S.


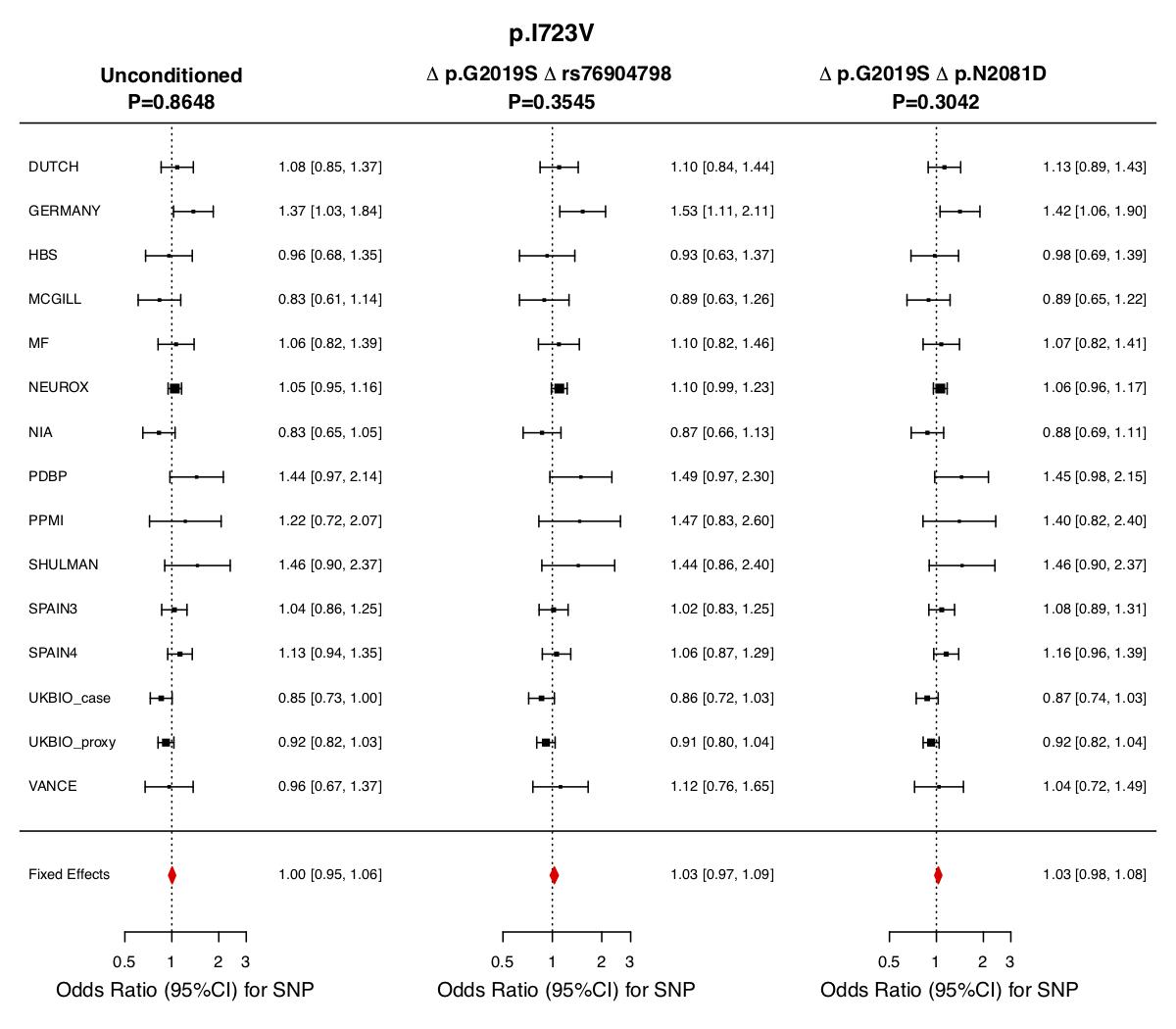


**Supplementary Figure 10.** Meta-analysis of p.I723V in the included datasets excluding (from left to right) 1) no samples 2) carriers of rs76904798 and p.G2019S and 3) carriers of p.N2081D and p.G2019S.


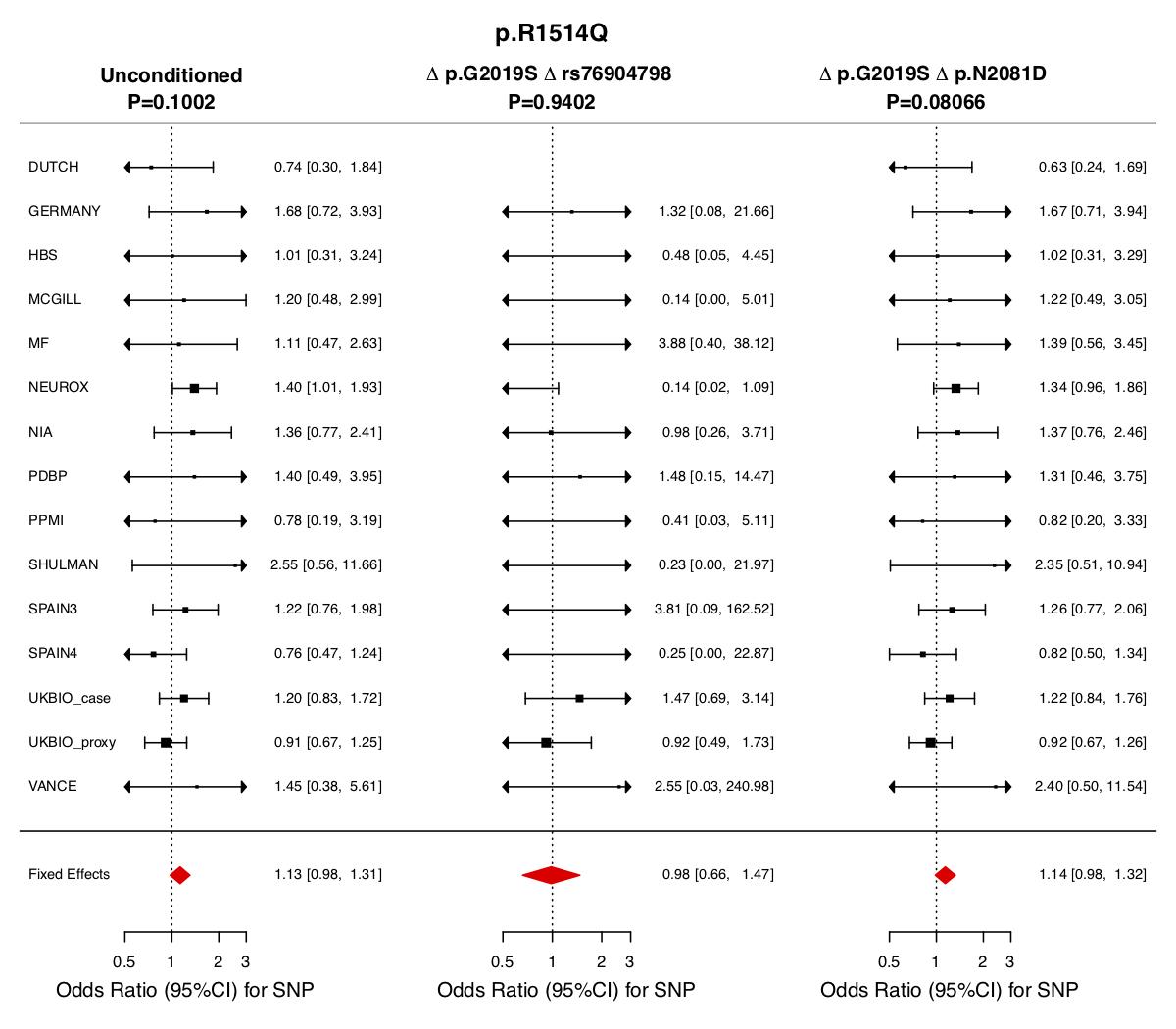


**Supplementary Figure 11.** Meta-analysis of p.R1514Q in the included datasets excluding (from left to right) 1) no samples 2) carriers of rs76904798 and p.G2019S and 3) carriers of p.N2081D and p.G2019S.

**
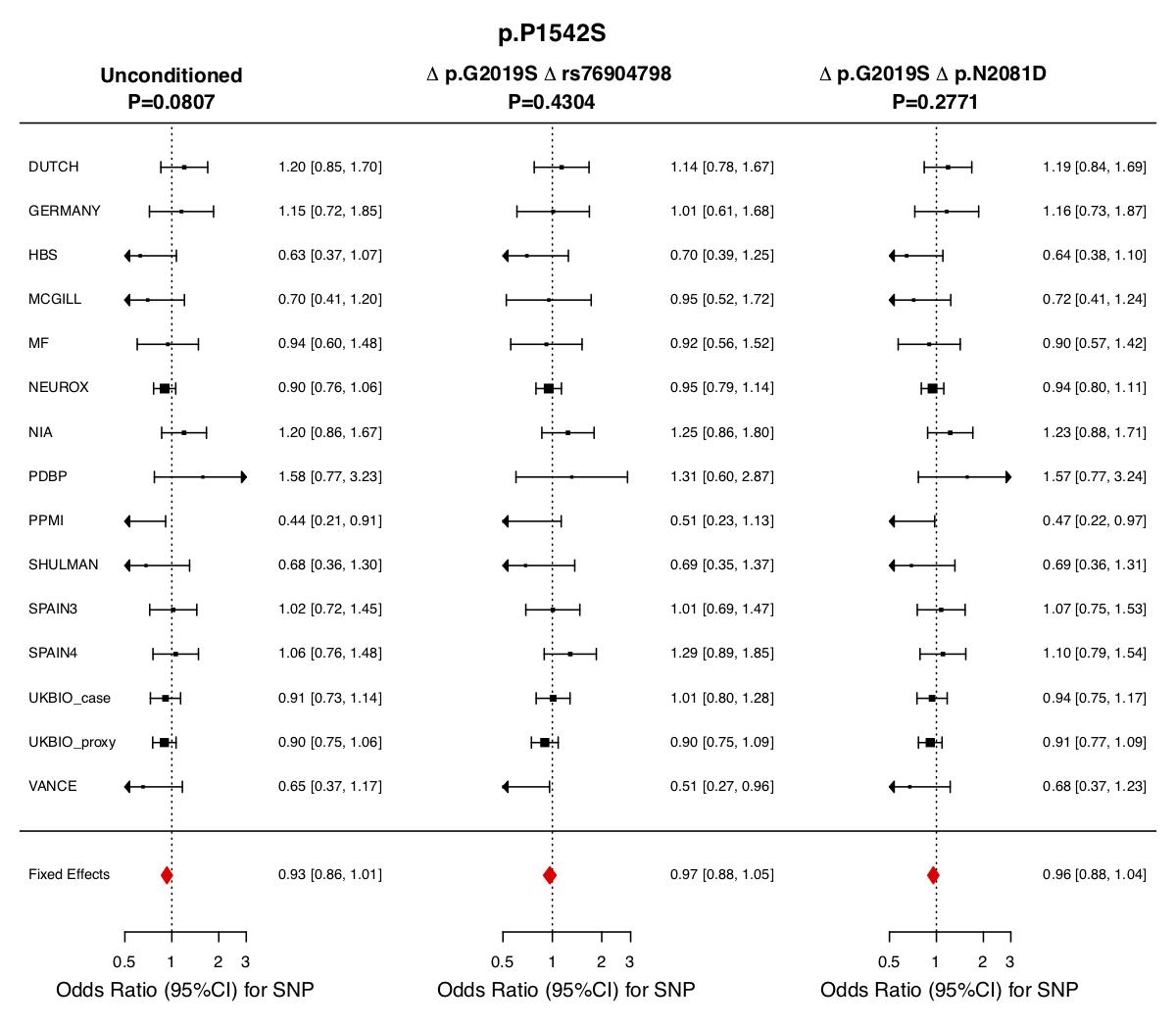
Supplementary Figure 12.** Meta-analysis of p.P1542S in the included datasets excluding (from left to right) 1) no samples 2) carriers of rs76904798 and p.G2019S and 3) carriers of p.N2081D and p.G2019S.


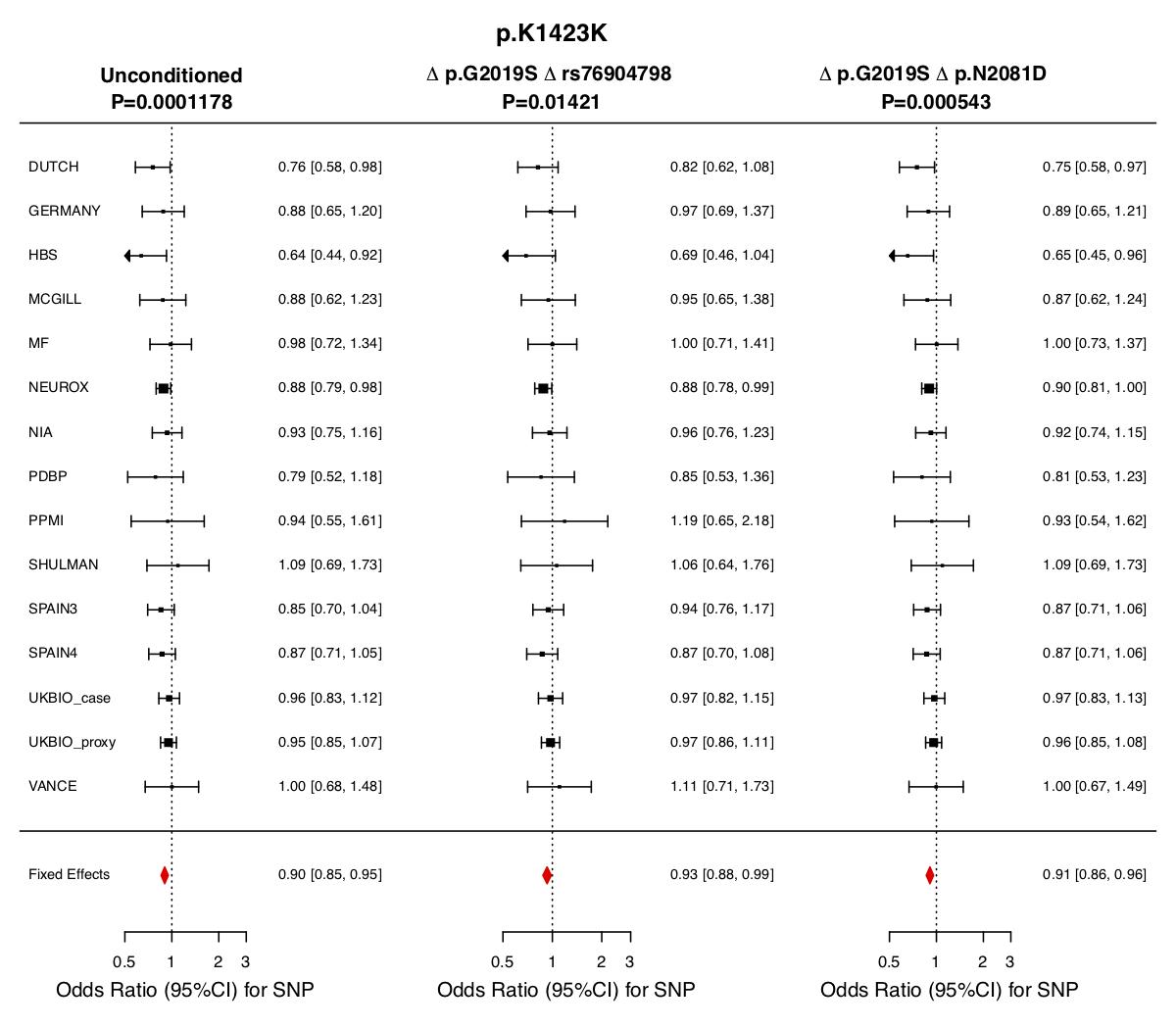


**Supplementary Figure 13.** Meta-analysis of p.K1423K in the included datasets excluding (from left to right) 1) no samples 2) carriers of rs76904798 and p.G2019S and 3) carriers of p.N2081D and p.G2019S.


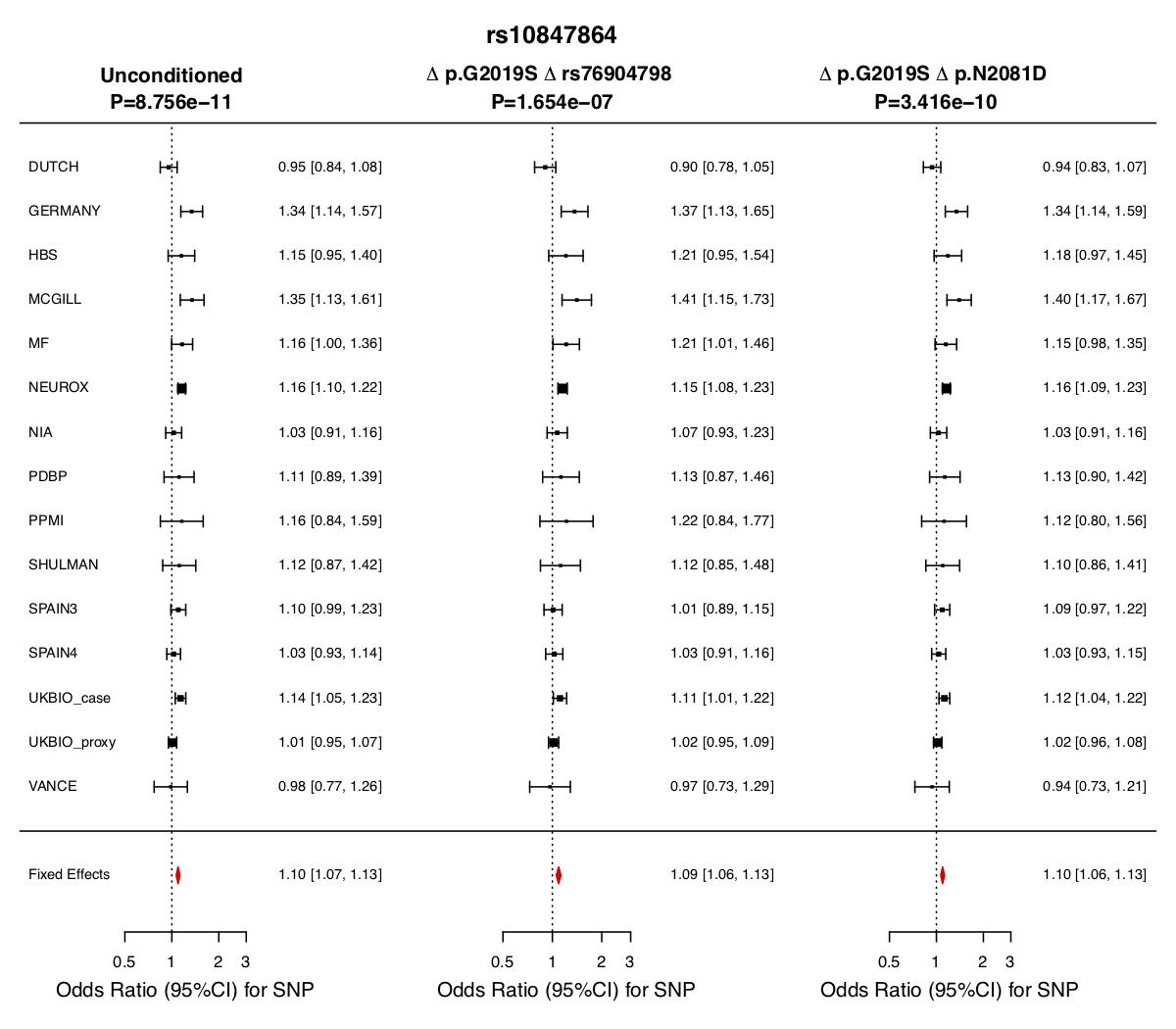


**Supplementary Figure 14.** Meta-analysis of rs10847864 in the included datasets excluding (from left to right) 1) no samples 2) carriers of rs76904798 and p.G2019S and 3) carriers of p.N2081D and p.G2019S.


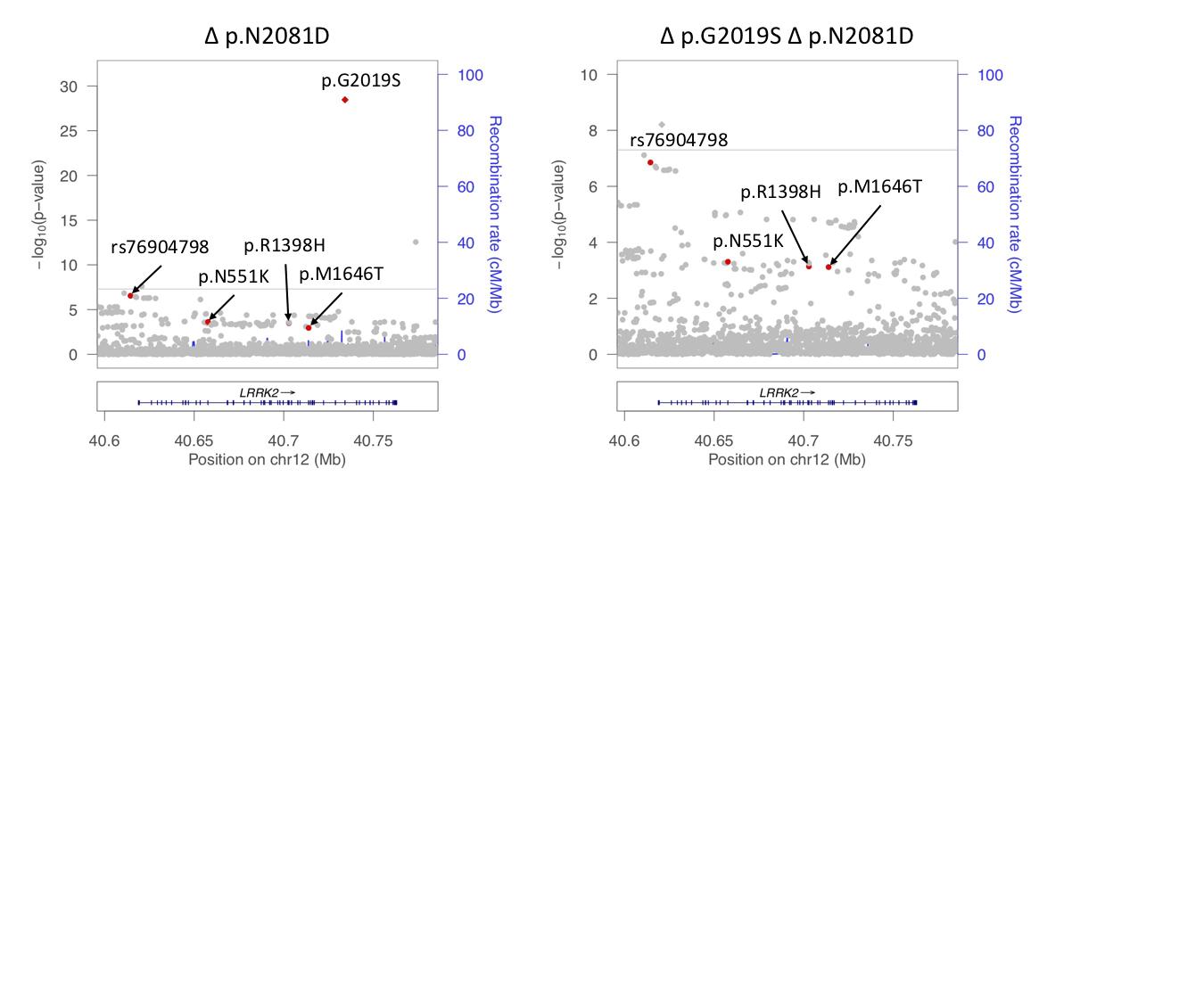
**Supplementary Figure 15.** LocusZoom plot of *LRRK2* association with Parkinson’s disease risk conditioned on p.N2081D. The left panel shows the association signal at the *LRRK2* locus in the IPDGC and UK Biobank meta-analysis conditioned on p.N2081D, and the right panel conditions on both p.G2019S and p.N2081D. The LRRK2 variants p.N551K, p.R1398H, p.M1646T, p.G2019S and rs76904798 are indicated by red dots.
